# Additive mixed meta‐analysis with spline meta‐regression for nonlinear ecological trends

**DOI:** 10.1002/ecy.70543

**Published:** 2026-11-05

**Authors:** Perry de Valpine, Melissa M. Foley, Marcus W. Beck, David B. Senn

**Affiliations:** ^1^ Department of Environmental Science, Policy and Management University of California Berkeley California USA; ^2^ San Francisco Estuary Institute Richmond California USA; ^3^ Tampa Bay Estuary Program St. Petersburg Florida USA

**Keywords:** ecological trend analysis, meta‐analysis simulation study, nonlinear meta‐analysis, nonparametric meta‐analysis, phytoplankton monitoring, regime change detection, San Francisco Bay water quality, spline meta‐analysis

## Abstract

Ecologists use meta‐analysis models to propagate uncertainty from statistical models of independent datasets into a higher‐level analysis. Meta‐analysis can be used to combine independent studies or as a two‐stage method for hierarchical modeling of data components from a large‐scale or long‐term study that are impractical to analyze in a single statistical model. While linear meta‐regression against study‐level covariates is common, ecological patterns are frequently nonlinear. For such cases, meta‐analysis methodology lacks tools to estimate semiparametric smooth meta‐regression models. We propose new spline meta‐regression methods by melding meta‐analysis with additive mixed models, resulting in the class of additive mixed meta‐analysis (AMMA) models. In a simulation study, when first‐stage SEs are heterogeneous, meta‐splines perform better than naive approaches such as simple generalized additive models (GAMs). In a case study to investigate 43‐year patterns of seasonally averaged phytoplankton levels in San Francisco Bay, California, USA, meta‐splines give notably different results than naive methods in such heterogeneous cases. Seasonal averages and SEs are estimated annually, then combined with meta‐splines to investigate long‐term, nonlinear changes in the season‐scale averages. The meta‐analysis approach is practical, fully propagates the statistical information and uncertainties, and provides advantages for visualization. The methods are available in the R package splinemixmeta.

## INTRODUCTION

Meta‐analysis comprises methods for analyzing data that are the results of previous analyses of separate data sets (Cooper et al., 2019). For example, one might have estimates of an ecological effect from many independent studies, each of which was analyzed separately. Meta‐analytic interest then lies in pooled effect sizes or in relationships of effect sizes to study‐level explanatory variables. Alternatively, one might have a large‐scale or long‐term study from which some ecological quantity is estimated independently for each year, site, and/or species. This might be done because the sampling protocol has changed over time, or for logistical reasons, or simply because components of the data are collected independently. In this case, meta‐analytic interest would lie in trends or patterns of the component estimates. Both problems involve two stages of analysis, where uncertainties from the first stage should be propagated to the second stage, which is the key feature of meta‐analysis. In this paper, we extend meta‐analysis methods to allow smooth nonlinear trends using the kinds of semiparametric regression approaches common to generalized additive models (GAMs). This combination may naturally be called additive mixed meta‐analysis, or AMMA. The methods presented here are available in the R package splinemixmeta (de Valpine & Beck, 2026).

Meta‐analysis regression (or *meta‐regression*) is common, so our extension to semiparametric approaches has many possible applications. For example, recent meta‐analyses include relating impacts of wildlife trade to travel time to human settlements (Morton et al., 2021), relating large herbivore impacts on ecosystems to body mass and soil properties (Trepel et al., 2024), relating bird migratory connectivity to distance and traits (Fattorini et al., 2023), relating island effects on body size to mainland body size (Benítez‐López et al., 2021), and relating public engagement with bird species to traits (Stoudt et al., 2022). In many such cases, the response variable is itself an effect size, so explanatory variables are called effect “moderators.”

While those examples highlight that meta‐analysis data often originate from separate studies, many researchers have recognized that meta‐analysis is also a tool for hierarchical modeling of large‐scale or long‐term data, as in our examples below. In that case, components of large data sets can be statistically modeled separately before being combined. For example, Dominici et al. (2000) and Samoli et al. (2008) used study‐level random effects for meta‐regression of coefficients relating public health to air pollution from multiple city‐level studies. A related use of meta‐analysis for hierarchical modeling is for integrated modeling of different kinds of data from the same study system. For example, Conn et al. (2022) combined multiple spatio‐temporal models, each from different data, into one model. In population dynamics, Knape et al. (2013) showed how to combine estimates with uncertainties from each year into a state‐space time‐series model. Notably, neither of the last two examples framed the approaches as “meta‐analysis,” although both qualify as such when a general view is taken.

In simple cases, such as estimating pooled effect sizes and SEs, meta‐analysis can be done using basic distribution theory without an explicit model to combine studies. That path leads into varieties of weighting schemes and estimands of interest (Borenstein et al., 2010). In more general cases, one needs a model for the relationship between study‐level covariates and outcomes. Study‐level random effects are appropriate to represent that each study has its own conditions and measures a quantity somewhat different than other studies (DerSimonian & Laird, 1986; Konstantopoulos & Hedges, 2019; Sera et al., 2019). These considerations lead to mixed‐effects meta‐analysis, the framework we use here.

Mixed‐effects meta‐analysis takes the form of a linear mixed model (LMM), where the roles of random effects and residuals need careful interpretation and some variance parameters are known in advance (Dominici et al., 2000; Platt et al., 1999; Sera et al., 2019). Suppose the study‐level responses (e.g., estimated parameters, or treatment‐control effect sizes such as Hedges' g or log response ratios) will be linearly regressed on the study‐level explanatory variables. Each study's response—even if perfectly known—may deviate from the regression trend. In basic regression, this would be residual variation because it occurs at the level of a single datum, but in meta‐analysis it is a random effect for the study. Additionally, each study's response is not known perfectly but rather is “measured” noisily by the first‐stage study. The SE of a first‐stage response is an estimate of the SD of this measurement error and thus represents the (now known) residual SD for that datum in the meta‐analysis model. Thus, a meta‐analysis LMM has multiple variance components, with one component's parameter (residual SD) assumed known for each datum. Note that purely fixed‐effects meta‐analysis is not of interest here because it is not realistic in the scenarios of this paper.

Despite that meta‐analysis models are placed within the common LMM framework, they are not just trivial applications of that framework. Different issues of estimation and inference arise (Follmann & Proschan, 1999; Gurevitch & Hedges, 1999; Viechtbauer, 2005; Yang et al., 2024). And, on the practical side, it is not typically easy in LMM software to allow datum‐specific variance parameters and to hold them fixed in advance. For this and other reasons, meta‐analysis is implemented in dedicated software packages such as the R packages mixmeta (Sera et al., 2019) and metafor (Viechtbauer, 2010).

Many ecological patterns and trends are nonlinear, so an important need for meta‐regression is to estimate nonlinear models without necessarily making strong assumptions about the form of nonlinearity. Here we meld the frameworks of meta‐analysis LMMs and smoothing splines as used in GAMs to achieve this. The known SEs from the first‐stage analyses provide a baseline level of variation that contributes to estimation of how smooth versus wiggly a spline should be. In the *Discussion*, we comment on three related approaches that could be used for some aspects of nonlinear meta‐analysis.

Mathematically, the frameworks of GAMs and meta‐analysis LMMs fit naturally together. Several approaches are commonly used for estimating GAMs with a key goal of avoiding overfitting. Here we use restricted maximum likelihood (REML) because it works naturally in the meta‐analysis context where some variance component parameters (residual SD for each datum) are known in advance. Extracting the fitted spline requires care because the REML approach handles splines as random effects. Predictors for random effects in LMMs are “BLUPs,” or best linear unbiased predictors, given other estimated parameters. In the cases of interest here, obtaining the spline BLUPs requires conditionally estimating some levels of random effects but not others. For implementation, we explain how the particular needs of AMMA formulation and estimation make it unsupported in common software tools (of which we are aware) and show how to combine tools and repurpose their features to achieve our goals.

We look at the performance of AMMA with simulations and then demonstrate its application for estimating long‐term, nonlinear trends of phytoplankton abundance (as indicated by chlorophyll‐*a* levels) in San Francisco Bay, California, USA. For simulations, we used scenarios with a regime shift from a high level to a low level over time, as might be driven by management interventions or changes in climate conditions. We generated different patterns and amounts of heterogeneity in first‐stage SEs and different relative magnitudes of variance components. We compared AMMA to several simpler methods that might be considered and show that AMMA is most useful when there is substantial heterogeneity in first‐stage SEs, whereas when these are homogeneous it performs similarly to a standard GAM, as one would expect.

The case study from San Francisco Bay is motivated by multi‐scale analysis of long‐term trends in water quality. Specifically, we asked whether season‐scale averages (e.g., fall averages with SEs produced by year‐specific statistical models, yielding one value and its SE per year) show trends across 43 years of data. While understanding finer‐scale dynamics is also of scientific interest, ecologists commonly recognize that multiple scales are relevant for understanding a system, and here our goal was to propagate uncertainties from season‐scale averages to multi‐year nonlinear patterns. This is important because at smaller time scales there can be higher frequency fluctuations, whereas seasonal averages can capture longer‐term trends. Importantly, there is no simple or straightforward way to make a unified analysis of the full time series incorporating both the within‐year components and the across‐year smoothing of seasonal averages; AMMA provides a way to achieve the goals of this multi‐scale analysis.

## MIXED‐EFFECTS META‐ANALYSIS

The mixed‐effects meta‐analysis framework for linear models is outlined by Sera et al. (2019), whose notation we follow, and the associated mixmeta R package (Gasparrini & Sera, 2025). We will refer to the analyses from original studies as *first‐stage* and the meta‐analysis as *second‐stage*. We describe the models and an overview of the methods here, while more technical details can be found in Appendix S1.

The m parameters estimated from the study i are in the vector $y_{i}$, for i=1…n studies (or portions of a long‐term study such as the years of monitoring data below). The $y_{i}$ are the response vectors of the meta‐analysis model, given by:

(1) $$y_{i}=X_{i}\beta +Z_{i}b_{i}+\epsilon_{i},\;i=1\ldots n,$$

where $X_{i}$ is the matrix of study‐level covariates and β the meta‐analysis coefficients, $Z_{i}$ is the random‐effects design matrix, $b_{i}˜N\left(0,\Psi_{i}\right)$ is the vector of random effects, and $\epsilon_{i}˜N\left(0,S_{i}\right)$ is the vector of residuals. For example, if each study yields three parameters, these compose the meta‐analysis response vector ($y_{i}$), with m=3. Equation (1) is a standard formulation of an LMM, where studies represent the more general notion of groups that may share random effects. In the simple case that the elements of $y_{i}$ should share a random intercept because they are from the same study, $Z_{i}$ would be a column of ones, $b_{i}$ would be a scalar, and $\Psi_{i}$ would be its variance across the n studies. Although we will be considering only scalar response variables of interest, we will need the full multivariate setting of Equation (1) later to incorporate a spline into the model.

The $S_{i}$ are inputs. These are the estimated covariance matrices of the $y_{i}$ from each of the n first‐stage analyses. They represent the variance of “measurement error” due to estimating parameters from study i, which in the context of study i in isolation would be called the “sampling variance,” that is, the variance of parameter estimates. In the case of a scalar parameter (m=1), $S_{i}$ is the SE squared. Typically, such as in a maximum likelihood or Bayesian analysis, $S_{i}$ is estimated by approximating the likelihood surface or posterior distribution, respectively, as Gaussian. This should often be a good approximation due, for example, to large‐sample likelihood theory. Thus plugging $S_{i}$ into the second‐stage analysis amounts to retaining a good approximation of the estimation uncertainty from the first stage.

The $\Psi_{i}$ remain to be estimated. For m=1, often one would assume $\Psi_{i}=\Psi$ for all i, that is, there is a shared random effects variance, an assumption of homoscedasticity. For m>1, one can assume $\Psi_{i}$ to be parameterized by a small number of parameters, such as in a temporal autoregressive or spatial autocorrelation structure.

Although the above has described the arrangement of independent studies as groups in an LMM framework, in fact arbitrary levels of nestedness can be handled. If some studies contain nested designs, such as treatment and control replication at each of multiple sites, this structure can be embedded in the $S_{i}$ and $\Psi_{i}$. Moreover, one can view all studies within a single outermost group, with a single S and single Ψ each composed as a block diagonal matrix containing the $S_{i}$ and $\Psi_{i}$ as blocks, respectively. In the steps below, we will define such an outer group to include all studies and include a spline random‐effects covariance matrix with correlations across groups along a covariate. Put more simply, since a spline for a nonparametric smooth term will be set up as a vector random effect, all data in the model must share that random effect, so we conceive of the “groups” for that random effect as comprising a single group with all the data.

## ADDITIVE MODELS

As our aim is to bring together mixed‐effects meta‐analysis and additive models in a common framework, we next introduce additive models. For response data $w=\left(w_{1},\ldots ,w_{n}\right)$, a normal (non‐generalized) additive model takes the form:

(2) $$w_{i}=\sum_{j=1}^{r}f_{j}\left(x_{i}\right)+\epsilon_{i}$$

where $x_{i},\;i=1\ldots n$, are covariate vectors, $f_{j}\left(\cdot \right),\;j=1\ldots r,$ are smooth functions of $x_{i}$ (each depending on one or a subset of covariates in $x_{i}$), and $\epsilon_{i}˜N\left(0,\sigma^{2}\right),\;i=1\ldots n$ are independently, identically distributed residuals (e.g., Marra & Wood, 2011; Wood, 2017). For example, if w is modeled as a smooth function of $x_{1}$ plus a smooth function of $x_{2}$ and $x_{3}$, Equation (2) would be $w_{i}=f_{1}\left(x_{1,i}\right)+f_{2}\left(x_{2,i},x_{3,i}\right)+\epsilon_{i}$.

Of primary interest are flexible functions for $f_{j}$ such as splines that can fit reasonably arbitrary smooth patterns in data but must be estimated with some regularization scheme to avoid overfitting. It is also common to include some functions that are assumed to be linear rather than flexible. In a case with some linear components and one spline, we would have

(3) w=Xβ+Zb+ϵ

Here X and β are (as above) a model matrix and coefficients, respectively, allowing both continuous and categorical predictors, just as in multivariate regression. Compared to Equation (1), w here represents all the data as a single group. Now Z is a matrix of spline basis functions evaluated at covariate values, and b is a vector of spline coefficients. (Sometimes the covariate used to calculate Z is a column of X, in which case one ensures that the basis functions do not redundantly represent a linear term in that covariate. Alternatively, one can allow the basis functions to include the linear term and omit the corresponding column from X to avoid redundancy.) Many splines use knots, a set of covariate values in the range of interest that define the maximum wiggliness that can be supported. In practice, one does not try to determine smoothness by the number of knots but rather chooses sufficiently many knots so that wiggliness will be determined by regularization rather than the specific choice of knots (Wood, 2017). A connection to the meta‐analysis model in (1) is immediately evident from (3) and will be made explicit in the next section.

There are many ways to estimate this model with some kind of regularization using a penalty on wiggliness to enforce smoothness. The REML method dovetails with mixed‐effects meta‐analysis and performs well compared to others (Marra & Wood, 2011), so we pursue it in this paper. See Appendix S1 for details.

## ADDITIVE MIXED META‐ANALYSIS (AMMA)

Now we are ready to combine mixed‐effects meta‐analysis and additive models to define additive mixed meta‐analysis. As a special case, this provides univariate nonparametric meta‐analysis, which is used in the example below. The similarities between the meta‐analysis model in (1) and the LMM representation of a spline in (3) suggest how the two frameworks can be merged.

We will suppose there is a single spline term for a scalar response estimated from each of the n studies. More general cases readily follow the same ideas as in this case. Define $y=\left(y_{1},\ldots ,y_{n}\right)$ to be the vector of study‐level responses and $s=\left(s_{1}^{2},\ldots ,s_{n}^{2}\right)$ their estimated variances, that is, squared SEs. As commented below (1), we can view all of y as a single outermost group in an LMM. The model is:

(4) $$y=X\beta +Z_{1}b_{1}+Z_{2}b_{2}+\epsilon$$

Here, the first random effects term, $Z_{1}b_{1}$, is a spline formulation. $Z_{1}$ contains the spline basis function evaluations at covariate values. The spline coefficients, $b_{1}$, are random effects following a multivariate normal distribution known up to one parameter, which is estimated by REML and determines the appropriate smoothness versus wiggliness of the spline.

The second random effects term, $Z_{2}b_{2}$, gives a vector of independent study‐level deviations (residuals) from the smooth curve (i.e., from $X\beta +Z_{1}b_{1}$). $Z_{2}$ is a design matrix, which in this case is simply $Z_{2}=I$, the identity matrix. The random effects $b_{2}$ are normally distributed with variance $\gamma_{2}$, that is, $b_{2}˜N\left(0,\gamma_{2}I\right)$. Clearly $Z_{2}$ could be removed from the notation in this case, but retaining it highlights the connection to the LMM format and also that one could easily include additional terms that group studies by other factors or covariates of interest. Sera et al. (2019) describe in detail how different kinds of meta‐analysis questions can be fit within the general framework, and here one can see that any of these can be used in the AMMA framework as well.

The “residual” term, ϵ, represents “measurement” error from the input studies, with known SDs $s_{j}$. Hence $\epsilon \sim N\left(0,S\right)$, where S is diagonal with $s_{j}^{2}$ as its j‐th diagonal element.

This model accomplishes the following. There is a smooth pattern in relation to the covariate entering the spline ($Z_{1}b_{1}$, where $Z_{1}$ is a function of the covariate). There are study‐level residuals ($b_{2}$) from that smooth pattern. And there is additional estimation error of known variance from each study (ϵ). The last term creates a lower bound on the amount of noise inherent in y, which (loosely speaking) creates an upper bound on the amount of wiggliness to be estimated via the spline. The examples below will illustrate these ideas.

Additional implementation details covered in Appendix S1 include the use of spline basis functions from mgcv as needed for REML estimation in mixmeta, as well as extracting the estimated spline components from the fitted mixmeta models.

## SIMULATION STUDY

We investigated several aspects of AMMA performance using combinations of data simulation scenarios and alternative analysis methods. The goals were to learn when and how meta‐analysis propagation of first‐stage uncertainty is important in practice. To address this, we considered four alternative methods that might be considered by a practitioner. First, we considered a simple GAM that ignores first‐stage uncertainty (i: “simple GAM”). If propagation of first‐stage uncertainty doesn't matter much, then a simple GAM should perform about as well as AMMA. Second, we considered naive (incorrect) use of a weighted GAM because that might easily be misunderstood as the right way to incorporate different SEs from first‐stage analyses (ii: “weighted GAM”). The weights were set to $1/s_{i}^{2}$, which retains the relative magnitudes of first‐stage variances but ignores among‐study heterogeneity beyond sampling error. The third method considered was a naive version of spline meta‐regression without the random effect for each study because one might imagine that this variance component is too much to worry about (iii: “naive meta”). Fourth and last, we included a method that represents an idealized upper limit on possible performance of AMMA (iv: “gold standard”). This is relevant because splines inherently have variable performance across the domain of their explanatory variables, and CI coverage may be inherently approximate. For the “gold standard” method, we used a spline with an idealized (impossible) dataset that has no estimation error, equivalent to SEs of zero from the first‐stage analyses. Thus, we fit each simulated data set to five models (AMMA and alternatives (i)–(iv)). These are described in more detail in Appendix S1.

The data simulation scenarios assumed a regime change over 40 years, starting with a mean value of 15, dropping gradually to 5, and stabilizing at 5. (The *x*‐axis could be any variable, not necessarily time.) While other true patterns would also be of interest for simulation, the regime‐change pattern appears to illustrate the key principles of when AMMA is most important compared to other methods, in addition to providing an example relevant to ecosystem change in response to large‐scale drivers over multiple decades. We focused simulation effort on different cases of first‐stage uncertainties rather than additional patterns of change.

Before considering any sources of variation, the mean value in year i is denoted $E\left[y_{i}\right]$, i=1,…,40. We assumed that for each of the 40 years, there is a random effect, $b_{i}˜N\left(0,\gamma \right)$, i=1,…,40. $b_{i}$ gives the actual deviation from the regime change pattern in year i. The first‐stage study in year i is thus trying to estimate $E\left[y_{i}\right]+b_{i}$. The first‐stage study collects $m_{i}$ independent, normally distributed data $y_{\mathrm{ij}}˜N\left(E\left[y_{i}\right]+b_{i},\sigma \right)$, $j=1,\ldots ,m_{i}$. The sample mean, $y_{i}=\sum_{j=1}^{m_{i}}y_{\mathrm{ij}}/m_{i}$, gives the $i^{\mathrm{th}}$ meta‐analysis response value. The sample SE squared, $s_{i}^{2}=\sum_{j=1}^{m_{i}}{\left(y_{\mathrm{ij}}-y_{i}\right)}^{2}/\left(m\left(m-1\right)\right)$, gives the $i^{\mathrm{th}}$ value of s used in the setup for Equation (4).

Simulation scenarios included different combinations of first‐stage residual variance and sampling effort. We considered three values of $\sigma^{2}$ increasing in accuracy: 20 (a: “less accurate”), 10 (b: “baseline”), and 5 (c: “more accurate”). The different values of $\sigma^{2}$ represent different magnitudes of residual variation in each first‐stage study.

For each value of $\sigma^{2}$, we considered multiple scenarios of first‐stage sampling effort. Sampling effort might vary across studies (years, in our example) due to unpredictable and/or systematic changes in resources. Sampling effort is used here as one reason that first‐stage SEs could be heterogeneous across studies. However, similar patterns could arise due to changes in measurement technology or for ecological reasons, such as different magnitudes of actual residual variation in different studies. The scenarios for sampling effort included constant effort (“C”: $m_{i}=5$ for all i, giving no heterogeneity), randomly varying effort (“R”: $m_{i}$ uniformly distributed from 2 to 8, independent across years), and a step‐change in sampling effort (“SC”: $m_{i}=2$ for the first half of the series, then $m_{i}=8$ for the second half). Randomly varying effort represents unpredictable research resources for each study, while the step‐change represents an increase in resources, perhaps in response to the possible trend. The constant (“C”) and random (“R”) sampling cases were used for all three values (a–c) of $\sigma^{2}$. To focus results, the step‐change (“SC”) sampling case was used only for the “more accurate” (c) value of $\sigma^{2}$.

For each value of $\sigma^{2}$ and relevant sampling effort scenarios (a–c), we simulated 500 data sets and estimated each of the five models described above. From those 500 replicates, we determined (for each of the five analysis models) as performance metrics the bias, variance, root‐mean‐squared‐error (RMSE), and CI coverage for each year's fitted values. We also demonstrate the visual interpretability gained by plotting first‐stage results within the second‐stage fitted model.

## CASE STUDY: SMOOTH CHLOROPHYLL‐ *a* TRENDS

We illustrate the semiparametric mixed‐effects meta‐analysis as a special case of AMMA using long‐term water‐quality monitoring data from SF Bay (Schraga & Cloern, 2017), as previously explored in Beck et al. (2022). The water‐quality variable of interest is a seasonal average of log chlorophyll‐*a* (chl‐*a*) concentration, which is an indicator of phytoplankton abundance. In this case, each separate “study” is really 1 year of data, which can be assumed independent across years. The first‐stage analysis for each year involves a spline fit to approximately biweekly (every 2 weeks) samples to estimate the average and its SE for a predefined season of interest while accounting for autocorrelation. It is merely a coincidence that the first‐stage analysis in this example also uses splines, completely unrelated to the meta‐analysis splines. In the second‐stage (meta‐) analysis, the goal is to estimate smooth variation across years of the within‐year seasonal averages while incorporating their uncertainties from the first‐stage analyses. In this case, year is the spline covariate. Although within‐year chl‐*a* dynamics are also of ecological importance, here interest centers on whether there are macroscopic (across‐year) trends in annual features such as seasonal averages.

Data were obtained from the U.S. Geological Survey San Francisco Estuary Research Program (Cloern & Schraga, 2016; Schraga & Cloern, 2017). This program collected water samples from 37 fixed locations (“stations”) along a 145 km longitudinal transect along SF Bay's deep channel from the southern extent of SF Bay to the lower Sacramento River. Surface water quality measurements available for each station include chl‐*a*, dissolved oxygen, photosynthetically active radiation (PAR), salinity, temperature, suspended particulate matter, and dissolved inorganic nitrogen, phosphorus, and silica. While the data record extends from 1968 to the present, methods and sampling intensity across stations became most consistent later, and we use data starting from 1977.

We chose to use data from three monitoring stations in the southernmost region (“Lower South Bay”) of SF Bay, stations 30, 32, and 36, each with 43 years of data (1977–2019). Chl‐*a* concentration is widely used as a water quality indicator, including in SF Bay, where changes in chl‐*a* concentration beginning in the late 1990s (Cloern et al., 2007) have focused attention on potential impacts of anthropogenic nutrient inputs to SF Bay (San Francisco Bay Regional Water Quality Control Board, 2012). The region's annual climate cycle includes distinct wet (Nov‐Apr) and dry (May‐Oct) periods, resulting in strong seasonal differences in physical forcings and biogeochemical responses, including quite different chl‐*a* levels and dynamics. Here we focus on the period from 1 August to 31 October (hereafter “fall”), a time window that captures seasonal fall phytoplankton blooms.

The three stations were chosen for the purposes of illustrating different kinds of results from meta‐splines. They are in a part of SF Bay with relatively higher levels of primary production and thus also represent interesting case studies. Based on results from the simulation study, we compare the meta‐spline (AMMA) results to “simple GAM” splines that ignore the estimated SEs of the first stage of analysis. This is both the easiest and most obvious of the alternative methods compared in the simulation study, so it is relevant to see how it compares to meta‐splines with real data.

The first‐stage analyses were done with the R package wqtrends (Beck et al., 2022, 2026), which used thin‐plate splines. For the second‐stage meta‐splines in the package splinemixmeta, we used cubic regression splines.

## RESULTS

### Simulation results

Incorporating first‐stage uncertainties into the second‐stage analysis using AMMA is most important when first‐stage uncertainties are heterogeneous (Table 1). In all cases where first‐stage uncertainties are homogeneous (“C,” left two columns in Table 1), the simple GAM (method (i)) and weighted GAM (method (ii)) both perform very similarly to AMMA. This result is expected because the assumption of homoscedasticity for the simple GAM is met, and the weights in the weighted GAM will be equal on average (varying only due to first‐stage stochasticity). The naive meta‐analysis (method (iii)) has a similar RMSE, but worse CI coverage, while the impossible “gold standard” (method (iv)) shows the best one can hope for.

**TABLE 1:** Simulation study results.

	RMSE	95% coverage	RMSE	95% coverage
(a) Less accurate case	constant (C) effort		random (R) effort	
AMMA (meta‐spline)	0.87	0.87	**0.84**	**0.88**
(i) Simple GAM	0.91	**0.89**	1.23	**0.88**
(ii) Weighted GAM	0.89	**0.89**	0.90	**0.88**
(iii) Naive meta	**0.86**	0.85	0.85	0.80
(iv) Gold standard	0.42	0.93	0.42	0.92
(b) Baseline case	constant (C) effort		random (R) effort	
AMMA (meta‐spline)	**0.65**	**0.92**	**0.68**	**0.92**
(i) Simple GAM	0.69	**0.92**	0.92	0.91
(ii) Weighted GAM	0.69	**0.92**	0.77	0.89
(iii) Naive meta	0.67	0.85	0.74	0.77
(iv) Gold standard	0.42	0.93	0.41	0.93
(c) More accurate case	constant (C) effort		random (R) effort	
AMMA (meta‐spline)	**0.55**	**0.93**	**0.57**	**0.93**
(i) Simple GAM	0.59	0.92	0.74	0.92
(ii) Weighted GAM	0.59	0.92	0.68	0.87
(iii) Naive meta	0.60	0.80	0.70	0.69
(iv) Gold standard	0.41	0.93	0.42	0.93
(c continued) More accurate case			step‐change (SC) effort	
AMMA (meta‐spline)			**0.54**	**0.93**
(i) Simple GAM			0.58	**0.93**
(ii) Weighted GAM			0.68	0.87
(iii) Naive meta			0.69	0.53
(iv) Gold standard			0.42	0.93

*Note*: There are three levels of mean first‐stage sampling error (a–c). Sampling effort is either constant (“C”; left two columns) or heterogeneous (either random, “R,” or step‐change, “SC”; right two columns). Results show root‐mean‐squared error (RMSE) and 95% confidence interval coverage for each of five methods: AMMA, three realistic but incorrect alternatives (i–iii), and an impossible “gold standard” (iv) assuming no first‐stage estimation error. Best results in each case are in bold.

Abbreviations: AMMA, additive mixed meta‐analysis; RMSE, root‐mean‐squared error; GAM, generalized additive model.

When first‐stage uncertainties are heterogeneous, due to either random (“R”) or step‐change (“SC”) sampling effort, AMMA produces lower (better) RMSE than all realistic alternatives (i–iii). When compared to the simple GAM (i), AMMA's improvement is larger when first‐stage uncertainties are larger. With small first‐stage uncertainties (c, “more accurate”) and random sampling effort (“R”), the RMSE from a simple GAM is 0.74, about 30% worse than 0.57 for AMMA. With “less accurate” (a) first‐stage results and random sampling effort (“R”), the simple GAM is more severely worse than AMMA, 1.23 compared to 0.84, or about 46% worse. When first‐stage uncertainties are heterogeneous due to a step‐change (“SC”) in sampling effort, and with the “more accurate” first‐stage results (“c continued”), the simple GAM performs less badly, only about 7% worse than AMMA (0.58 vs. 0.54). It makes sense that simple GAMs don't perform well with heterogeneous first‐stage uncertainties, because those result in heteroscedasticity and thus violate an assumption of the simple GAM.

Performance of the weighted GAM alternative (ii) is intermediate to the simple GAM and AMMA when sampling effort is random (“R”). This makes sense because the weighted GAM makes use of the different first‐stage SEs but does not also incorporate study (year) level variation as a random effect. However, in the case when sampling effort undergoes a step‐change (“SC”), the weighted GAM performs *worse* than the simple GAM.

Performance of the naive meta‐analysis alternative (iii) is also intermediate to the simple GAM and AMMA when sampling effort is random (“R”) and also worse than the simple GAM when sampling effort undergoes a step‐change (“SC”). Similarly to the weighted GAM, the naive meta‐analysis incorporates first‐stage SEs (but not in exactly the same way) but omits study (year) level random effects, so it is not surprising that these two methods perform similarly in terms of RMSE. Of course, with a real data set, one doesn't know which scenario is relevant, so one can't cherry‐pick an alternative as the best available incorrect method and would instead do better with AMMA in all cases.

The point‐wise CI coverage for AMMA is systematically less than nominal (values are <95%), but that is also the case for the “gold standard” (iv), illustrating that it is an issue with splines in general. Note that spline CI coverage is expected to be accurate only when averaged across a spline (Nychka, 1988) and will still be approximate for limited data. In comparison to the alternative methods, the simple GAM (i) tends to have similar coverage as AMMA, while the weighted GAM (ii) has slightly worse coverage and the naive meta‐analysis (iii) has much worse coverage.

When inspecting example simulated data sets to gain intuition, one sees that simple GAMs in some cases match AMMA closely while in other cases they clearly overfit the data (Figures 1, 2, 3). Each pair of examples gives a flavor of how different simulated data sets can give different comparisons between methods, sometimes with pronounced differences (left panels) and sometimes only minor differences (right panels). The worst overfitting is seen when first‐stage accuracy has a step‐change (Figure 3, left), resulting in spurious fluctuations in one half of the series. These figures illustrate the visualization benefit of a two‐stage analysis. One can see all the information from the first‐stage as point estimates with uncertainties and can interpret the AMMA fits intuitively. Figures showing the same examples with the other comparison methods are in Appendix S1.

**FIGURE 1:**
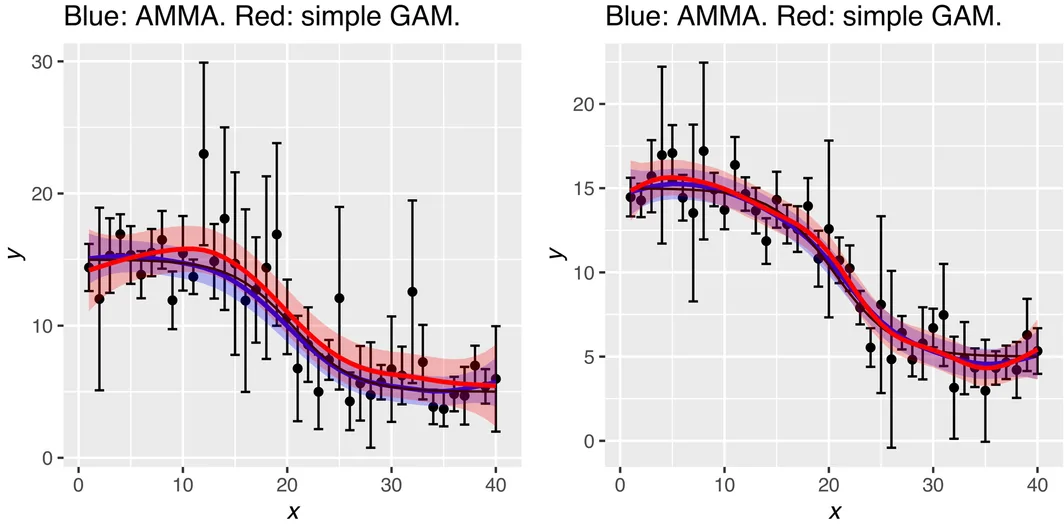
Two example simulated data sets with the “more accurate” (c) first‐stage errors and random (“R”) sampling effort. Points show first‐stage estimates with 95% confidence intervals. Mean y (e.g., log chl‐*a*) decreases from 15 to 5 over 40 values of x (e.g., year).The black line shows the correct model, from which data were generated. Blue shows the fit and 95% confidence interval (BLUP ±2 SEs) for additive mixed meta‐analysis. Red shows the same for a simple generalized additive model (GAM) (alternative method (i)). The left panel shows a case where the worse fit of the simple GAM is visually clear. The right panel shows a case where the two methods fit almost identically.

**FIGURE 2:**
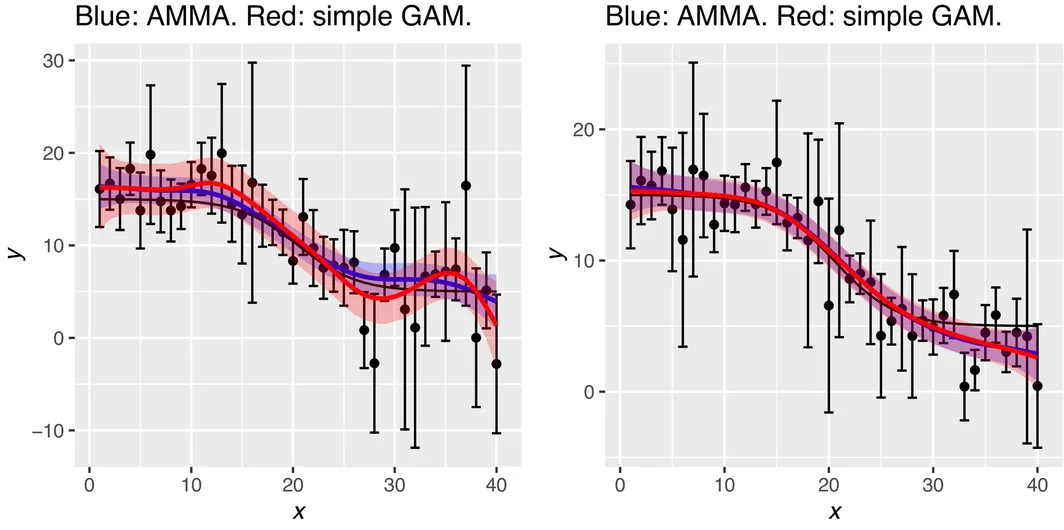
Similar to Figure 1, with “less accurate” (a) first‐stage errors and random (“R”) sampling effort. In the right panel, the blue line is nearly exactly overlaid by the red line.

**FIGURE 3:**
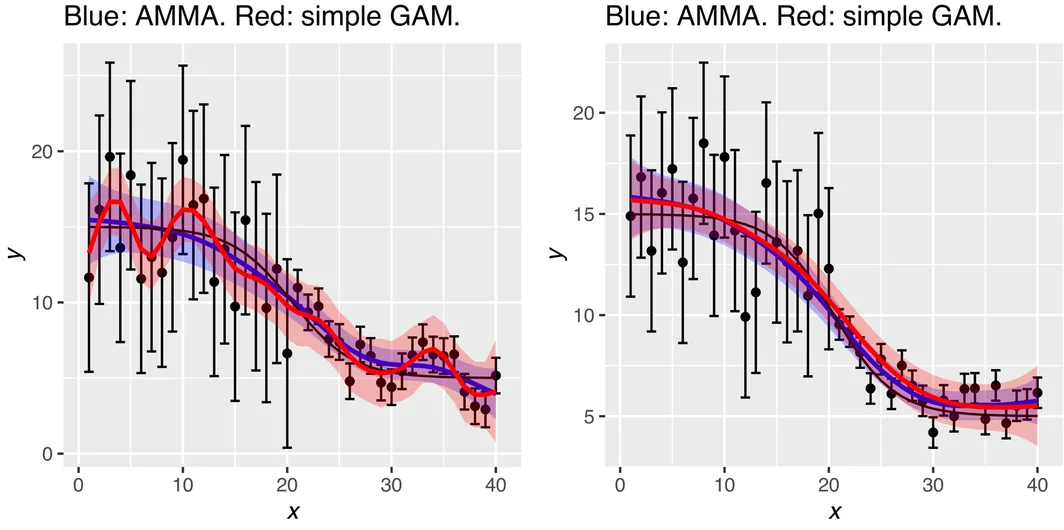
Similar to Figures 1 and 2, with “more accurate” (c) first‐stage errors and a step‐change (“SC”) in first‐stage sampling effort.

### Water quality monitoring results

The three examples of chl‐*a* annual results illustrate three different estimation phenomena. At station 30 (Figure 4), first‐stage uncertainties were fairly homogeneous (with a notable exception in 1981), and results from AMMA and a simple GAM are nearly identical. At station 32 (Figure 5), there are a few additional large first‐stage uncertainties. These increase the residual error of the simple GAM and result in it having a notably larger confidence envelope than AMMA. We interpret the AMMA result as better because it recognizes that some points are far off the curve due to their known large first‐stage SEs, allowing smaller uncertainties in the meta‐spline. As a result, the meta‐spline provides higher confidence that the decline from approximately 2008 onward is real.

**FIGURE 4:**
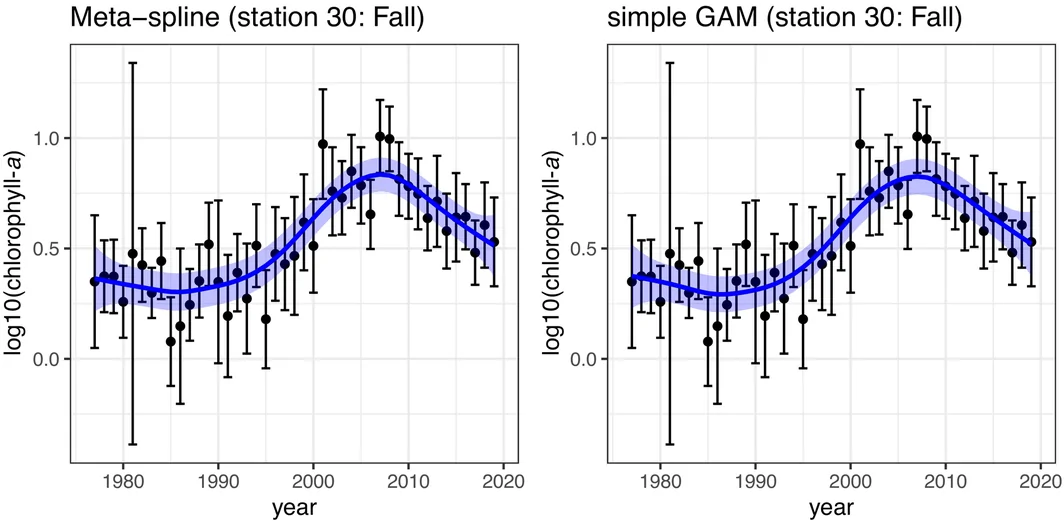
Meta‐splines (additive mixed meta‐analysis, left) and simple splines (right) for fall seasonal averages across years from station 30. BLUPs ±2 SEs are shown for the splines. In this case, the methods give nearly identical results.

**FIGURE 5:**
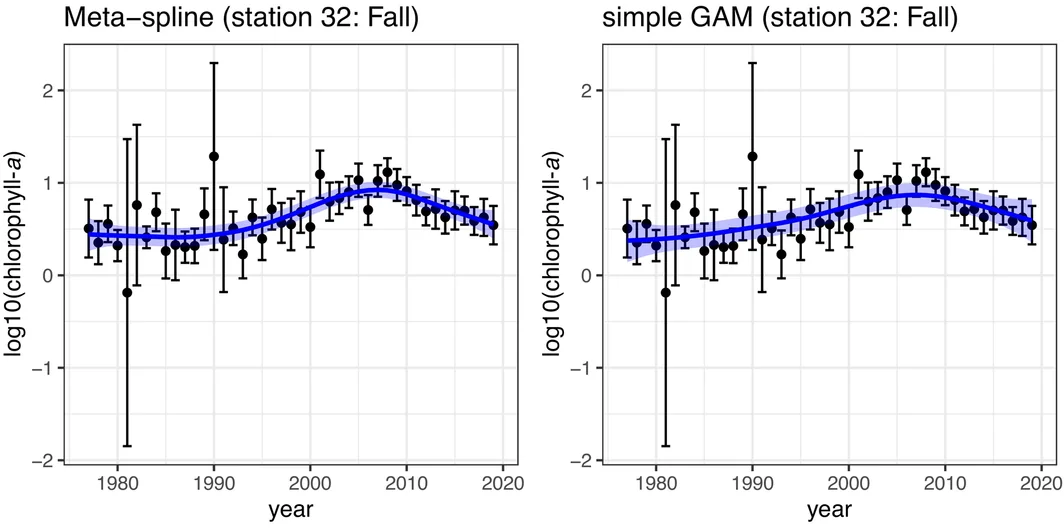
Meta‐splines (left) and simple splines (right) for fall seasonal averages across years from station 32. BLUPs ±2 SEs are shown for the splines. Meta‐spline give greater precision.

At Station 36 (Figure 6), there are even more very noisy first‐stage estimates, and they appear to throw the simple GAM into a bad case of overfitting. The estimated smoothness parameter is quite small because the large fluctuations early in the series, when their large uncertainties are ignored, necessitate a high degree of wiggliness. This leads to what we judge to be overfitting of the data later in the series too, where excessive wiggliness in the blue curve is visually evident. AMMA successfully avoids this kind of overfitting, resulting in a smoother curve and narrower confidence bands. In general, when looking at the data from station 36, one might reasonably question whether some of the data in early years should be excluded as outliers or too noisy to be useful, but low effort or noisy sampling occasions do occur in long‐term data, and it is desirable to have a method that is robust when including them.

**FIGURE 6:**
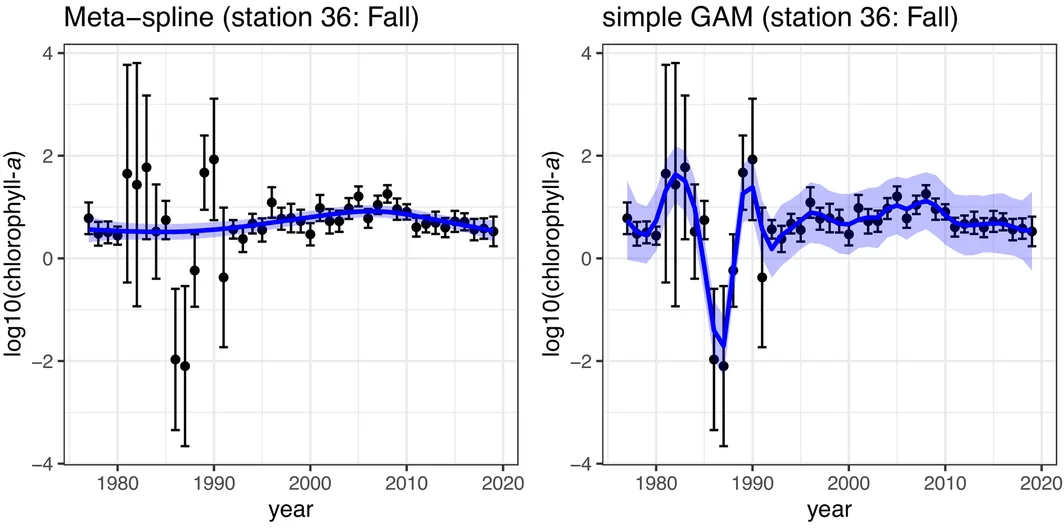
Meta‐splines (left) and simple splines (right) for fall seasonal averages across years from station 36. BLUPs ±2 SEs are shown for the splines. Simple generalized additive model (GAM) overfits to noisy early data.

## DISCUSSION

We have provided methods for semiparametric regression in a meta‐analysis framework. These methods will allow researchers to investigate potentially nonlinear patterns between study‐level results and study‐level explanatory variables, or moderators. As illustrated in our examples, these methods can also be used to estimate trends across time (or other variables) from long‐term or large‐scale ecological monitoring programs. In the San Francisco Bay example, we illustrated the use of these methods to achieve a multi‐scale analysis that would not be straightforward to do with simpler methods.

On a statistical level, we have extended mixed‐effects meta‐analysis models to include basis function expansions with regularization estimated by REML. Doing so allows estimation of nonlinear meta‐regression patterns as smooth functions such as splines. This provides the ingredients for a class of additive mixed meta‐analysis, or AMMA, models. Although the examples here use a single spline term and no fixed effects in the meta‐analysis, the framework presented naturally can support more complicated combinations of model terms.

Our approach contrasts favorably to three related approaches that could be used for some aspects of semiparametric meta‐analysis. First, it is already possible within existing meta‐analysis software, including metafor and mixmeta, to add specific covariance structures for random effects associated with study‐level covariates. For example, if each study takes place at a different time or place, one can add a random effects vector with an autoregressive or distance‐decay covariance structure, respectively. Our approach is more general for all the reasons that basis function approaches (including smoothing splines) are popular in GAMs. The models can be interpreted in terms of basis vectors and their coefficients, which have advantages such as dimension reduction, visualization, and examining collinearity with measured covariates (Hefley et al., 2017). A variety of useful basis functions are known and readily usable, such as cubic splines and cyclic splines (Wood, 2017). We note that thin‐plate splines were more difficult to use in this context. The basis function framework can also encompass Gaussian process models, such as for continuous spatial autocorrelation and Markov random fields, such as for discrete spatial units (Wood, 2025). Finally, estimation approaches have been well explored, and many software tools are available for creating the spline components needed for our purposes.

The second kind of related approach involves different ways to combine splines and meta‐analysis, with different goals. Some investigators have used a GAM or GAMM in the first‐stage analyses of each separate study, and then used linear mixed‐effects meta‐analysis to analyze the spline coefficients (or spline predicted values) across studies (Schwartz & Zanobetti, 2000; Sera et al., 2020). For example, spline coefficients from each study might represent the estimated mean value of a response at each of a set of covariate values of interest. Meta‐analysis might then look for linear across‐study patterns (including simply pooled estimates) in the within‐study nonlinear patterns (represented by spline coefficients). The methods here are different in that they look for nonlinear across‐study patterns in estimates from within each study. Orsini et al. (2022) took a step in this direction, but they limited second‐stage spline flexibility by setting a small number of knots rather than using more general regularization methods to let the data determine optimal smoothness.

The final set of related approaches uses a Bayesian framework and associated computational methods in one or both stages of analysis (Higgins et al., 2009; Johnson et al., 2022; McCaslin et al., 2021). This has advantages and disadvantages. In the frequentist methods used here, the parameter estimates and SEs from the first stage represent a Gaussian approximation to the log‐likelihood surface (or more generally to an estimation surface). That approximation will typically be accurate due to large‐sample likelihood theory, with the result that the second‐stage analysis will be very similar to a full joint analysis of all the data. However, if large sample theory is not accurate for the first‐stage models (i.e., if posteriors are not approximately Gaussian), and/or if first‐stage data are fit jointly and thus are not independently estimated, then Bayesian methods provide one way to fully represent the information from the first stage by using a Monte Carlo posterior sample. This does come with technical challenges if the second‐stage analysis needs information from the tails of the first‐stage posteriors, for which the posterior samples may not have many points. On the other hand, the reasons many people favor Bayesian methods, such as propagation of uncertainties into predictions, also remain relevant. Here we pursued frequentist methods as they were well‐suited to our purposes.

There are several possible directions for future improvements or alternative steps. It would be useful to investigate the estimation properties of meta‐spline methods, including RMSE and coverage accuracy of CIs, in a wider range of scenarios. With LMMs more generally, coverage may be only approximate if some levels of replication are not very high (Pinheiro & Bates, 2004). Notwithstanding this concern, a potential benefit of having mixed‐effects meta‐analysis and AMMA within the LMM framework is for model‐checking methods. Finally, one could consider bootstrapping or resampling‐based methods, ideally from the first‐stage data, which we have not pursued here. Given that large‐scale, long‐term, and multi‐study data sets are increasingly common, further development of flexible and robust meta‐analysis models and methods will be valuable for estimating nonlinear responses to climate drivers, gradual regime changes, and many other kinds of ecological questions.

## AUTHOR CONTRIBUTIONS

David Senn and Melissa Foley posed the questions of long‐term, multi‐scale trend analysis for San Francisco Bay, provided the USGS data, and interpreted its results. Perry de Valpine developed the methodology and simulation study. Marcus Beck provided the first‐stage analyses through wqtrends. Perry de Valpine and Marcus Beck created the splinemixmeta package. Perry de Valpine drafted the manuscript with input and revisions from all authors.

## CONFLICT OF INTEREST STATEMENT

The authors declare no conflicts of interest.

## Supporting information


Appendix S1.

## Data Availability

Data and code for the simulation study and San Francisco Bay example (de Valpine, 2026) are available in Zenodo at https://doi.org/10.5281/zenodo.19404095. Code for the methods (de Valpine & Beck, 2026) is available in Zenodo at https://doi.org/10.5281/zenodo.19402410. This is the source code for version 1.0.1 of R package splinemixmeta.
