# Supplementary material for: Additive mixed meta‐analysis with spline meta‐regression for nonlinear ecological trends
Source: Ecology. 2026 Nov 5;107(11):e70543. doi: 10.1002/ecy.70543 (PMC13740663; doi:10.1002/ecy.70543)
Supplement: Supplementary file 1 [file ECY-107-e70543-s001.pdf]

Appendix S1

Additive mixed meta-analysis with spline meta-regression for nonlinear ecological trends

Perry de Valpine, Melissa M. Foley, Marcus W. Beck, David B. Senn

Ecology

The methods presented in this paper are available in R package `splinemixmeta` (de Valpine and Beck, 2026). Code and data for the simulation study and San Francisco Bay example are archived on Zenodo (de Valpine, 2026).

S1 More details about the models and estimation methods

We start from the mixed-effects meta-analysis model, given by

$$\mathbf{y}_i = \mathbf{X}_i\boldsymbol{\beta} + \mathbf{Z}_i\mathbf{b}_i + \boldsymbol{\epsilon}_i, i = 1 \ldots n, \tag{S1}$$

This includes random effects $\mathbf{b}_i \sim N(\mathbf{0}, \boldsymbol{\Psi}_i)$, where the $\boldsymbol{\Psi}_i$ need to be estimated. The marginal covariance of $\mathbf{y}_i$ is $\text{Cov}(\mathbf{y}_i) = \boldsymbol{\Sigma}_i = \mathbf{Z}_i\boldsymbol{\Psi}_i\mathbf{Z}_i' + \mathbf{S}_i$. For $m > 1$, one can assume $\boldsymbol{\Psi}_i$ to be parameterized by a small number of parameters, such as in a temporal autoregressive or spatial autocorrelation structure. Restricted maximum likelihood (REML) can be used to estimate those parameters.

We next consider the connection to an additive model specified with some linear components and one spline, given in the main text by:

$$\mathbf{w} = \mathbf{X}\boldsymbol{\beta} + \mathbf{Z}\mathbf{b} + \boldsymbol{\epsilon}. \tag{S2}$$

Assume $\mathbf{b} \sim N(\mathbf{0}, \boldsymbol{\Psi})$ where $\boldsymbol{\Psi}$ is known up to a constant, γ, such that $\boldsymbol{\Psi}(\gamma) = \gamma\boldsymbol{\Psi}_0$, where $\boldsymbol{\Psi}_0$ is known. Then the model takes the form of a linear mixed effects model with spline basis function values as the random effects model matrix, $\mathbf{Z}$. The best linear unbiased predictor (BLUP) of $\mathbf{b}$, which is its conditional mean given the data (using estimated parameters), represents the estimated smooth curve (spline). The conditional covariance matrix of $\mathbf{b}$ can be used to obtain confidence regions around the BLUP, which can also include uncertainty from estimation of $\boldsymbol{\beta}$.

Choice of $\boldsymbol{\Psi}_0$ is revealed from the connection to other regularization methods such as generalized cross-validation (GCV; Wood, 2017). In general, one estimates $\boldsymbol{\beta}$ by maximizing a penalized log likelihood.

$$-\frac{1}{2\sigma^2}\left|\left|\mathbf{w} - (\mathbf{X}\boldsymbol{\beta} + \mathbf{Z}\mathbf{b})\right|\right|^2 - \lambda\mathbf{b}'\boldsymbol{\Omega}_0\mathbf{b} \tag{S3}$$

where λ controls the strength of penalization. The rationale for penalizing coefficients via $\mathbf{b}'\boldsymbol{\Omega}_0\mathbf{b}$, with known $\boldsymbol{\Omega}_0$ derived from the basis functions, is that this quantity is the sum of squared curvature (second derivative magnitudes) of the spline, representing its wiggliness (Wood, 2017, in whose notation $\mathbf{S}$ is $\boldsymbol{\Omega}_0$ here). The degree of penalization is controlled by λ, which (in the penalization context) must be selected outside of a likelihood framework, such as via GCV, which we do not discuss further. The connection to the random effects formulation is that $-\lambda\mathbf{b}'\boldsymbol{\Omega}_0\mathbf{b}$ takes the same form as the log likelihood contribution from $\mathbf{b}$ of the random effects model (S2), i.e. $-\mathbf{b}'(\gamma\boldsymbol{\Psi}_0)^{-1}\mathbf{b}/2$. Specifically, if $\boldsymbol{\Omega}_0$ has an inverse, then $\boldsymbol{\Psi}_0 = \boldsymbol{\Omega}_0^{-1}$ and $\lambda = 1/(2\gamma)$. Within additive model methodology, this connection can be viewed as a rationale for the use of REML to estimate the smoothness parameter λ (Wood, 2017). It is also a formulation used for a Gaussian process prior for $\mathbf{b}$ in a Bayesian context.

Two comments are important for implementation. First, one is free to rotate the $\mathbf{b}$ and correspondingly $\mathbf{Z}$ and $\boldsymbol{\Psi}_0$, without changing the marginal likelihood. This reparameterization allows one to diagonalize the penalty, which can be useful. Specifically, we can write $\boldsymbol{\Psi}_0 = \mathbf{Q}\boldsymbol{\Lambda}_0\mathbf{Q}'$, where $\mathbf{Q}$ is a matrix of eigenvectors (with $\mathbf{Q}^{-1} = \mathbf{Q}'$) and $\boldsymbol{\Lambda}_0 = \boldsymbol{\Lambda}_0^{1/2}\boldsymbol{\Lambda}_0^{1/2}$ a diagonal matrix of eigenvalues (with $\boldsymbol{\Lambda}_0^{1/2}$ a diagonal matrix of their

square roots). Define $\mathbf{b}_d = \mathbf{\Lambda}_0^{-1/2}\mathbf{Q}'\mathbf{b}$. Then the penalty term involves $\mathbf{b}'(\gamma\mathbf{\Psi}_0)^{-1}\mathbf{b} = \mathbf{b}_d'\mathbf{I}\mathbf{b}_d/\gamma$, where $\mathbf{I}$ is the identity matrix. Correspondingly, with $\mathbf{Z}_d = \mathbf{Z}\mathbf{Q}\mathbf{\Lambda}_0^{1/2}$, one has $\mathbf{Z}\mathbf{b} = \mathbf{Z}_d\mathbf{b}_d$.

Second, in the penalized likelihood formulation, $\mathbf{\Omega}_0$ need not be full rank. This allows $\mathbf{b}$ to be *unpenalized* in some direction(s) (the null space) of the parameter space (Marra and Wood, 2011; Wood, 2017). For example, a linear basis function can be included but unpenalized. In the random effects view, if the REML implementation will use numerical methods such as covariance matrix decompositions, these may require a proper full rank covariance matrix. In other words, they may require that $\mathbf{\Omega}_0^{-1}$ exists. Hence, care is needed when inter-operating existing software tools. In our case, these will include R packages `mgcv` (Wood, 2025) to obtain spline basis functions and penalty matrices and `mixmeta` (Gasparrini and Sera, 2025) to estimate mixed-effects meta-analysis models.

The last three terms in equation (4) of the main text can be interpreted as random effects versus residual terms in a couple of ways. Viewing the spline term as of primary interest, the last two terms can be interpreted as two residual components. In that view, the total residual covariance is $\gamma_2\mathbf{Z}_2\mathbf{\Psi}_2\mathbf{Z}_2' + \mathbf{S} = \gamma_2\mathbf{I} + \mathbf{S}$. (Here for generality we use $\mathbf{b}_2 \sim N(\mathbf{0}, \gamma_2\mathbf{\Psi}_2)$. In the simple univariate case, both $\mathbf{Z}_2$ and $\mathbf{\Psi}_2$ are the identity matrix, $\mathbf{I}$. $\mathbf{S}$ is assumed known from first-stage analysis in the mixed-effects meta-analysis framework.) On the other hand, the middle two terms are both random effects terms. Viewed this way, the total residual covariance is only $\mathbf{S}$, and the total random effects covariance is $\gamma_1\mathbf{Z}_1\mathbf{\Psi}_1\mathbf{Z}_1' + \gamma_2\mathbf{Z}_2\mathbf{\Psi}_2\mathbf{Z}_2'$.

S2 Implementation Details

The main implementation challenges involve obtaining spline basis functions and the associated penalty matrix and using REML estimation with known residual variance structure. For splines, because there are many kinds of basis functions, it is appealing to use existing spline implementations in a black-box manner, such that any basis functions that admit penalties that use a quadratic form as shown above can be used in the meta-splines. For this purpose we use the popular `mgcv` package. What is needed are $\mathbf{Z}$ and $\mathbf{\Psi}_0 = \mathbf{\Omega}_0^{-1}$. For REML estimation of a mixed-effects meta-analysis model that can handle many kinds of additional random effects structures (beyond those used in our example), we use the `mixmeta` package.

A difficulty in inter-operating `mgcv` and `mixmeta` is that `mgcv` provides $\mathbf{\Omega}_0$, and routinely it is not full rank. This means that unpenalized directions such as a linear term are included in the spline and that $\mathbf{\Omega}_0^{-1}$ does not exist. In order to obtain $\mathbf{\Psi}_0 = \mathbf{\Omega}_0^{-1}$ for use in the REML implementation of `mixmeta`, one must move any null space components in $\mathbf{\Omega}_0$, and correspondingly in $\mathbf{b}$, out of the spline basis and (equivalently) into the fixed effects part of the model. Such directions can be identified as the eignevectors that have an eigenvalue of 0 in the diagonalized versions of $\mathbf{\Omega}_0$ and $\mathbf{Z}$ ($\mathbf{Z}_d$ above). The `smoothCon` and `smooth2random` functions of `mgcv` makes it easy to obtain these. One must be careful to include those components as fixed effects, i.e. as part of $\mathbf{X}\boldsymbol{\beta}$, so that they are not omitted from the model entirely. (The `splinemixmeta` package allows this to be done automatically or manually, controlled by the `manual_fixed` argument.)

Another implementation difficulty involves how to extract the estimated spline coefficients, $\mathbf{b}_1$ (referring to equation (4) of the main text). In theory, these can be obtained as the best linear unbiased predictors (BLUPs), i.e. the conditional means given the data, using the parameters estimated by REML and including only the spline portion of the random effects terms. However, in implementation such as via package `mixmeta`, extracting BLUPs using one random effect but not others can be tricky. In this case, one does not want BLUPs to include $\mathbf{b}_2$ as random effects but rather as residuals. The `splinemixmeta` package provides versions of the `predict` and `blup` functions to make this easier.

We also note a trick that can be useful for obtaining BLUPs for some random effects but not others. The model can be re-written as:

$$\mathbf{y} = \mathbf{X}\beta + \mathbf{Z}_1\mathbf{b}_1 + \boldsymbol{\epsilon}'. \tag{S4}$$

where $\boldsymbol{\epsilon}' \sim N(0, \mathbf{S} + \gamma_2\mathbf{I})$. After γ_2 has been estimated using REML, $s_i^2 + \gamma_2$ is the known variance of y_i. Hence REML can be used to estimate $\boldsymbol{\beta}$ and γ_1 using (S4), with $\mathbf{S} + \gamma_2\mathbf{I}$ known, and the resulting estimates of $\boldsymbol{\beta}$ and γ_1 must be identical to those from the primary AMMA formulation. Now the random effects BLUPs from (S4) will represent the spline term of interest.

S3 Details of the simulation models

Data sets for the simulation study were generated as follows. Let $x_i = i$ be the year, for $i = 1 \ldots 40$. Define $\tilde{x_i}$ as x_i transformed by subtracting the mean and dividing by the standard deviation, i.e. centering and scaling the years. The true underlying pattern of regime change was

$$E[y_i] = 5 + 10 \times \text{expit}(-4\tilde{x}_i) \tag{S5}$$

where expit(x) = 1/(1+exp(x)). This pattern for $E[y_i]$ gives a sigmoidal decline from 15 to 5 over 40 time steps.

For first-stage study i, we assumed the random effect is $b_i \sim N(0, \gamma)$ (this is b_{2i} in eq. 4 in the main text). Thus the first-stage analysis for study i (using data at x_i) is trying to estimate $E[y_i] + b_i$.

We assumed that first-stage study i estimates $E[y_i] + b_i$ as the sample mean from m_i independent data points, $y_i = \sum_{j=1}^{m_i} y_{ij}/m_i$, with $y_{ij} \sim N(E[y_i] + b_i, \sigma)$, $j = 1, \ldots, m_i$. The sample standard error squared is $s_i^2 = \sum_{j=1}^{m_i}(y_{ij} - y_i)^2/(m(m-1))$, which is used in equation (4). The estimates y_i and s_i^2 are part of $\mathbf{y}$ and $\mathbf{S}$, respectively, in the meta-analysis.

Note that we did not actually have to simulate the m_i values of y_{ij}. Instead, we used the facts that:

- $y_i \sim N(E[y_i] + b_i, \sigma^2/m)$;
- $\sum_{j=1}^{m_i}(y_{ij} - y_i)^2/\sigma^2 \sim \chi^2_{m-1}$;
- y_i and $\sum_{j=1}^{m_i}(y_{ij} - y_i)^2$ are independent.

. Thus we simulated y_i and s_i^2 directly, without using draws of y_{ij}. Specifically:

- $b_i \sim N(0, \gamma)$;
- $y_i = E[y_i] + b_i + \epsilon_i$, where $\epsilon_i \sim N(0, \sigma^2/m)$;
- $s_i^2 = \frac{\sigma^2}{m(m-1)}\nu_i$, where $\nu_i \sim \chi^2_{m-1}$.

For simplicity we always used $\gamma = 1$. The choices of σ^2 are meaningful relative to that value.

In general, any more complicated first-stage analysis, including Bayesian methods, might have produced y_i and s_i; our use of the sample mean from independent data points is just for simplicity.

S4 Comparison models for the simulation study

The five models used for the simulation study included AMMA, three models easily conceivable but wrong as attempts to incorporate first-stage measurement error, and a gold-standard model that uses better data than the others. For the spline term, all models (including the AMMA model), used cubic regression splines with 15 knots.

- AMMA: This model is given in the main text.

  The R call to fit the AMMA models was:

  ```
  splinemixmeta::splinemixmeta(s(x, bs = "cr", k = 15), y ~ x,
  se = se, manual_fixed=TRUE)
  ```

- Simple GAM: This model is given by

  $$\mathbf{y} = \mathbf{X}\boldsymbol{\beta} + \mathbf{Z}\mathbf{b} + \boldsymbol{\epsilon} \tag{S6}$$

  where $\mathbf{Z}$ and $\mathbf{b}$ are defined identically as for the AMMA model, and $\boldsymbol{\epsilon} \sim N(0, \sigma^2\mathbf{I})$ is a vector of independent, normal residuals. The differences between the simple GAM and the AMMA model are that all residuals have the same variance and that there is no separation between a residual term and a sampling variance from first-stage standard errors. If first-stage standard errors are nearly homogeneous (i.e., homoscedastic), the simple GAM will often give very similar results to AMMA.

The R call to fit the simple GAM models was:

```
mgcv::gam(y ~ s(x, bs = "cr", k = 15))
```

- Weighted GAM: This model is the same as for the simple GAM, but with a different residual distribution. The weighted residual distribution is $\epsilon_i \sim N(0, \sigma^2 s_i^2)$. Here the variance parameter σ^2 still needs to be estimated, and the variance for y_i is inversely scaled by the weight $1/s_i^2$. Thus, where the AMMA model assumes the variance for y_i around the fixed effects and spline term is $\gamma_2 + s_i^2$, the weighted GAM assumes it is $\sigma^2 s_i^2$. This model should also give results very similar to AMMA if first-stage standard errors are nearly homogeneous.

  The R call to fit the weighted GAM models was

```
mgcv::gam(y ~ s(x, bs = "cr", k = 15), weights = 1/se^2)
```

- Naive meta-analysis: This model is the same as the AMMA model (eq. 4) in the main text but with the $\mathbf{Z}_2\mathbf{b}_2$ term removed. This assumes that the *only* source of deviation from the mean pattern (spline) is the first-stage measurement error. Thus, instead of $\gamma_2 + s_i^2$, the naive meta-analysis uses simply s_i^2 as the variance of y_i, with no parameter to estimate.

  The R call to fit the naive meta-analysis was

```
splinemixmeta::splinemixmeta(s(x, bs = "cr", k = 15), y ~ x,
se = se, manual_fixed=TRUE, residual_re=FALSE)
```

- Gold standard: The gold standard model was a simple GAM fit to better data than the other models used. This used values of y_i with no first-stage measurement error, i.e. $\epsilon_i = 0$ in the simulation summary above (or, equivalently, all $m_i \to \infty$). This means that the residual variance in the simulations is γ, and the simple GAM is the correct model. This model is useful to reveal the best-case scenario for AMMA if first-stage standard errors are extremely small. For example, confidence interval coverage may still not be perfect due to properties of GAMs with limited data.

  The R call to fit the gold-standard model was the same as for the simple GAM above, but with the idealized (less noisy) version of `y`.

S5 Additional results from simulation study

The main text includes figures from two example data sets from each of three scenarios, showing the AMMA model and the simple GAM for comparison. Each pair of figures was chosen to illustrate one example where the fits clearly differ and one where they are very similar. Here (figures S1-S9) we include similar figures showing the other three comparison methods for the same example data sets. Each figure has the same result for AMMA and a different comparison method. There may be other example data sets that illustrate poor versus good results for each comparison method; the ones shown here are the same examples as used in the main text to illustrate AMMA compared to simple GAM and thus do not necessarily fully illustrate results from each method.

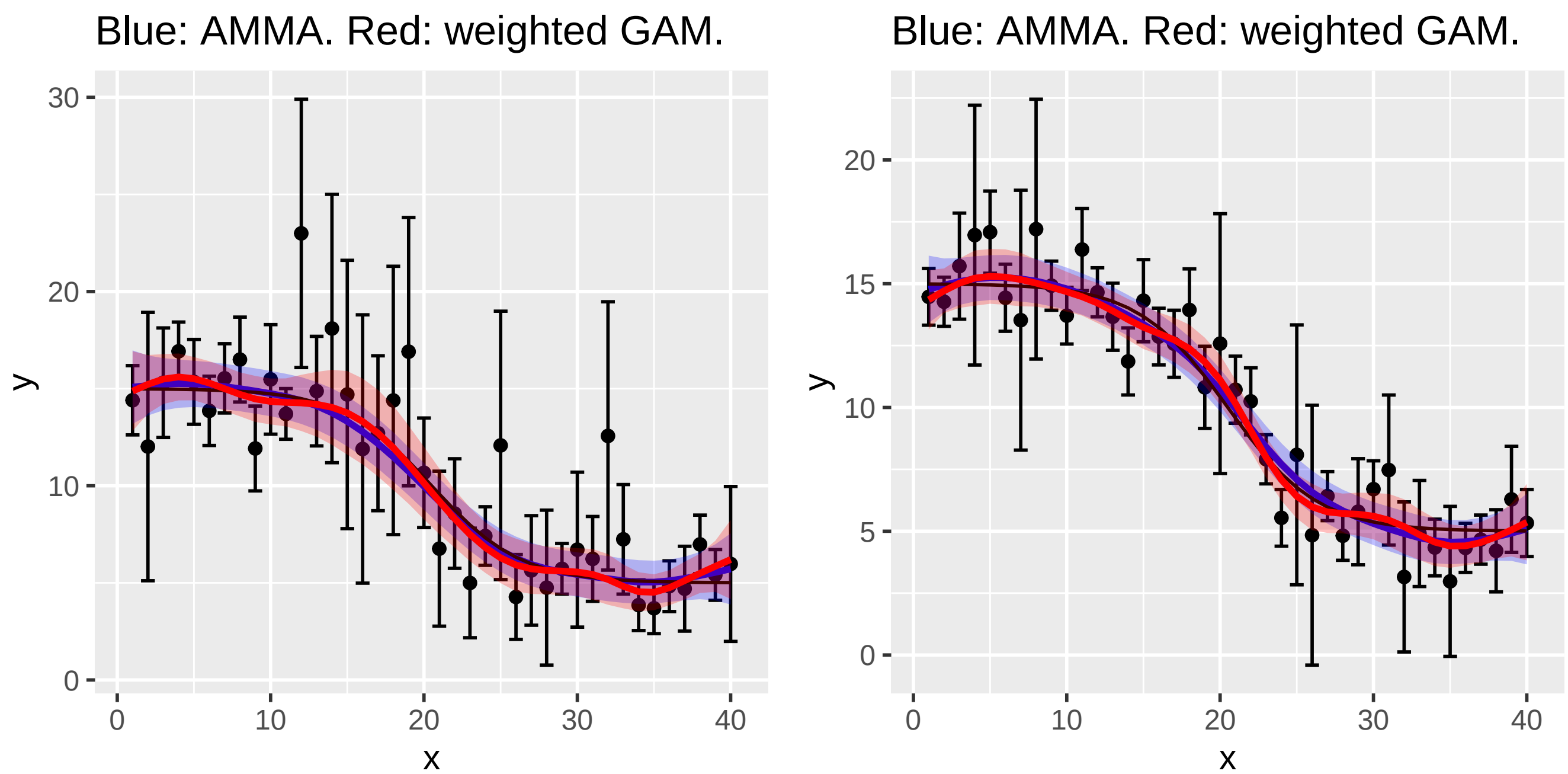

Figure S1: Examples with “more accurate” (c) first-stage errors and random (“R”) first-stage sampling effort, comparing AMMA to weighted GAM.

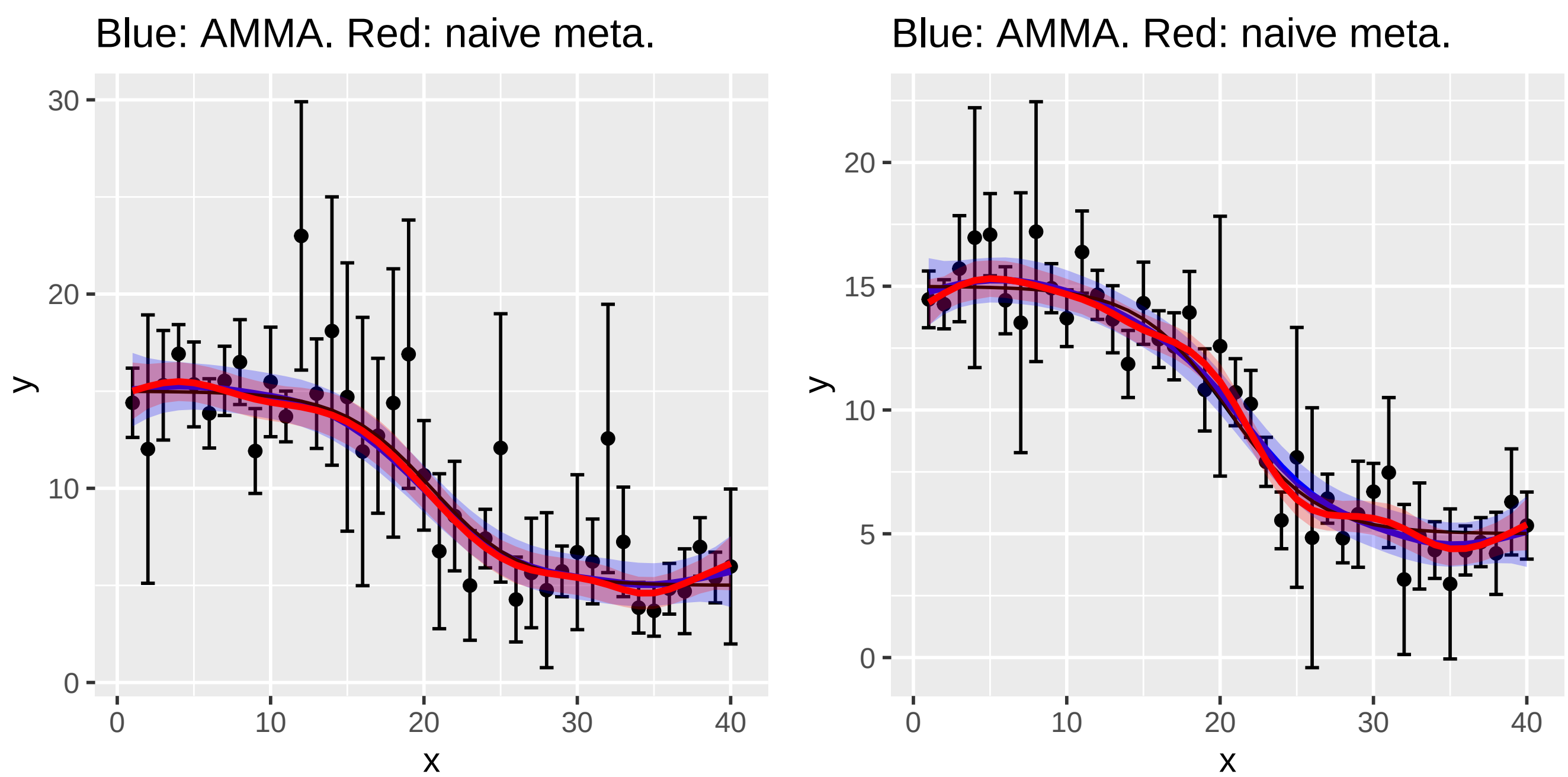

Figure S2: Examples with “more accurate” (c) first-stage errors and random (“R”) first-stage sampling effort, comparing AMMA to naive meta-analysis.

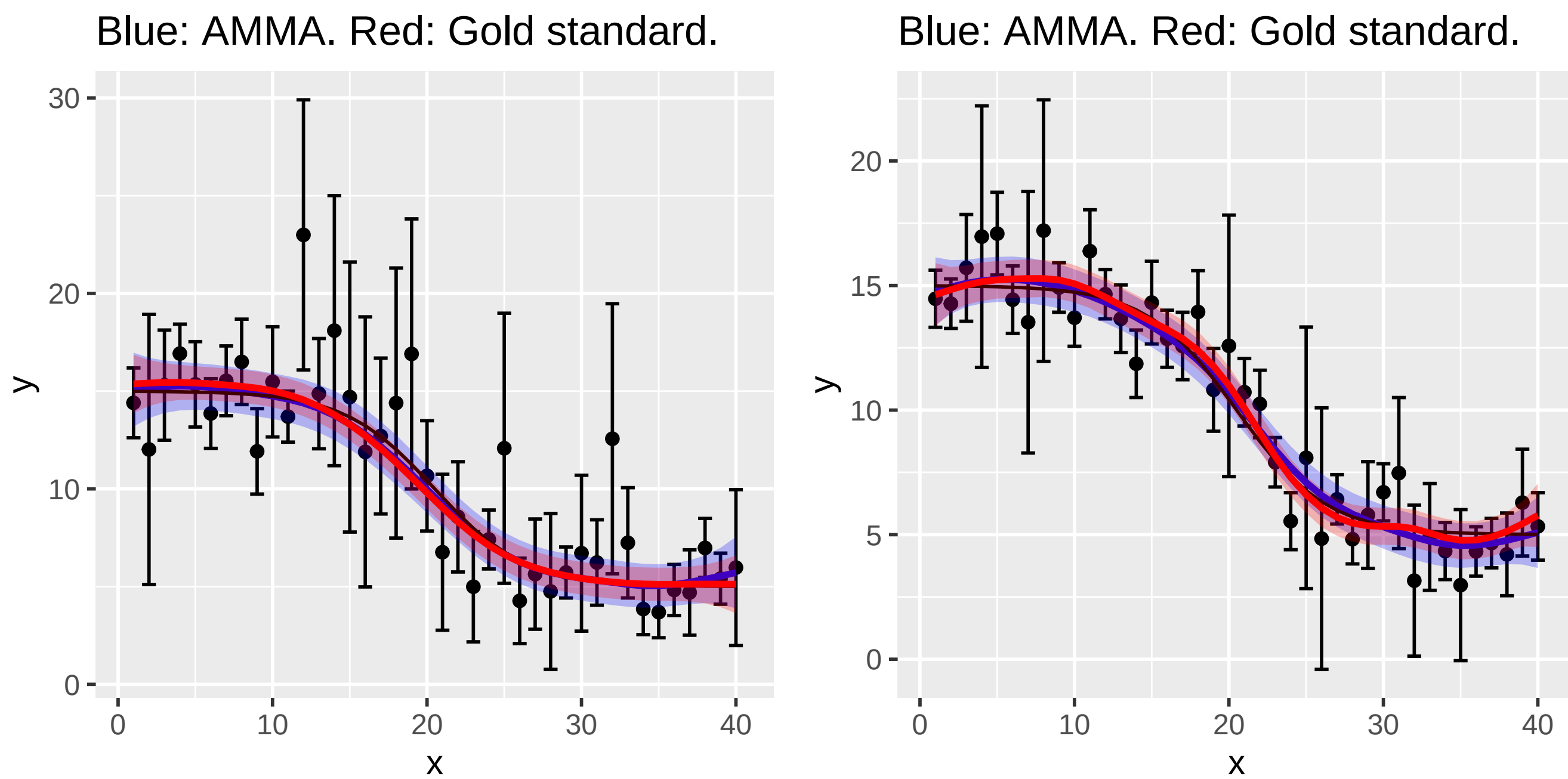

Figure S3: Examples with “more accurate” (c) first-stage errors and random (“R”) first-stage sampling effort, comparing AMMA to gold standard.

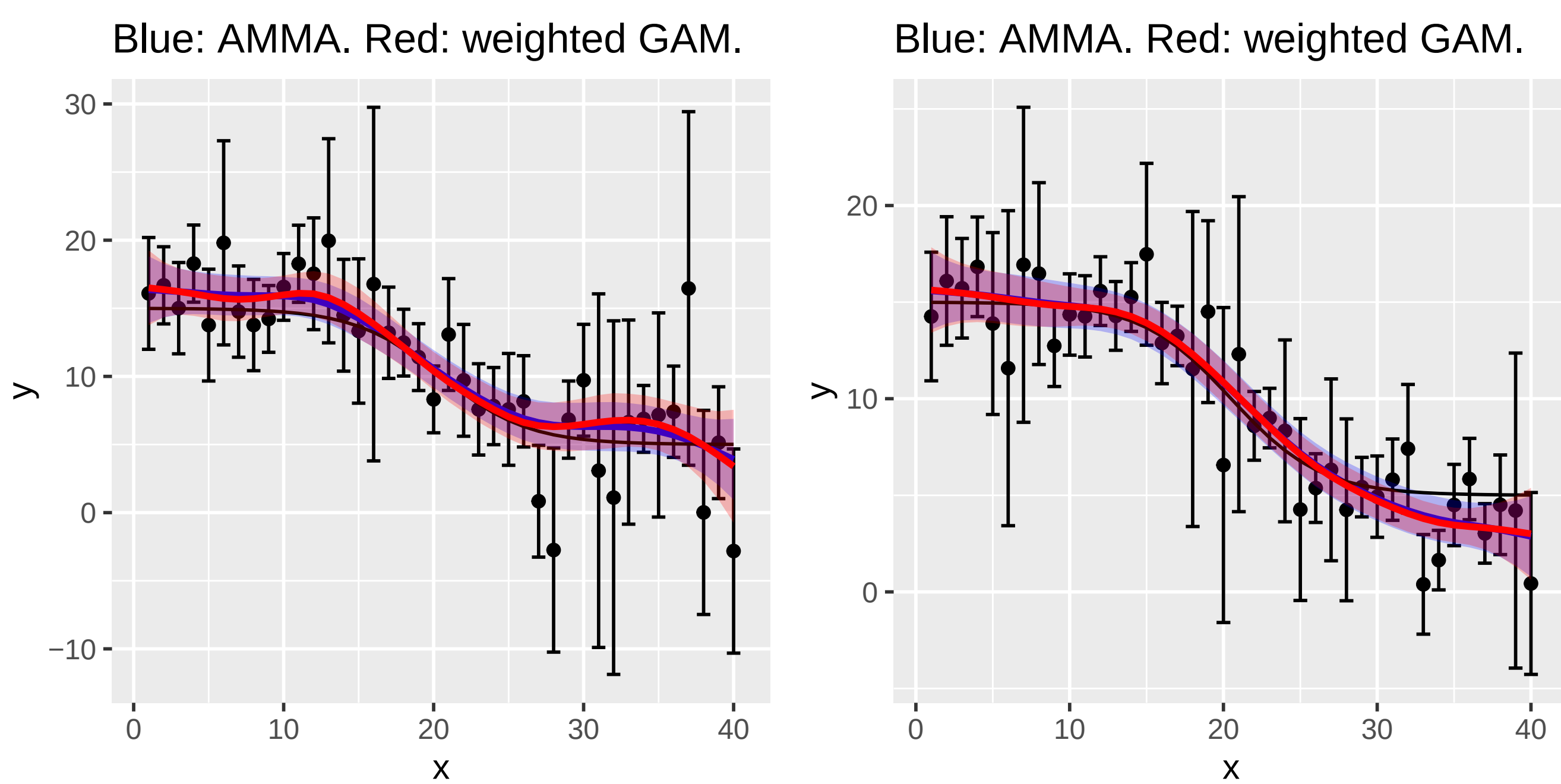

Figure S4: Examples with “less accurate” (c) first-stage errors and random (“R”) first-stage sampling effort, comparing AMMA to weighted GAM.

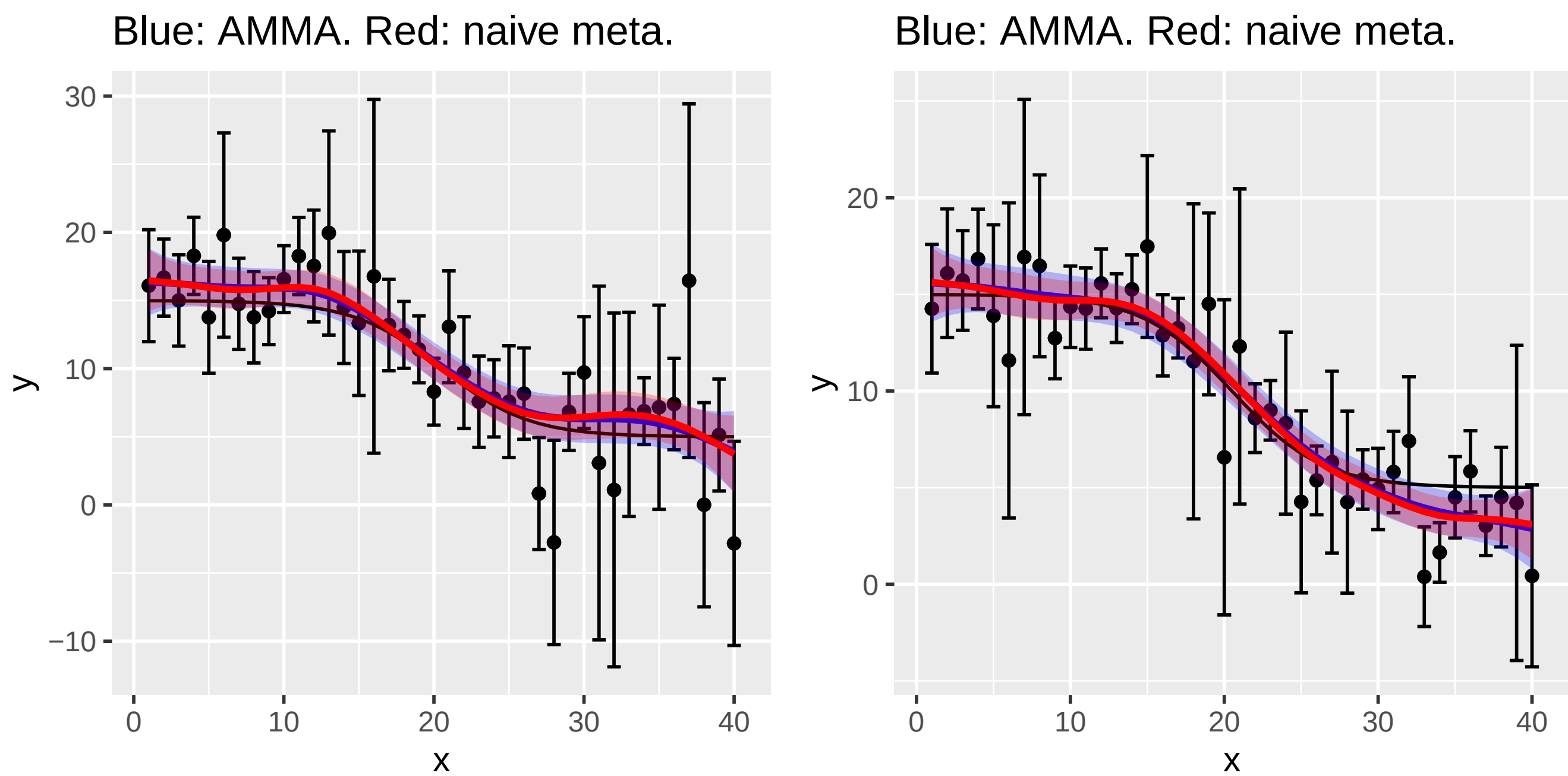

Figure S5: Examples with "less accurate" (c) first-stage errors and random ("R") first-stage sampling effort, comparing AMMA to naive meta-analysis.

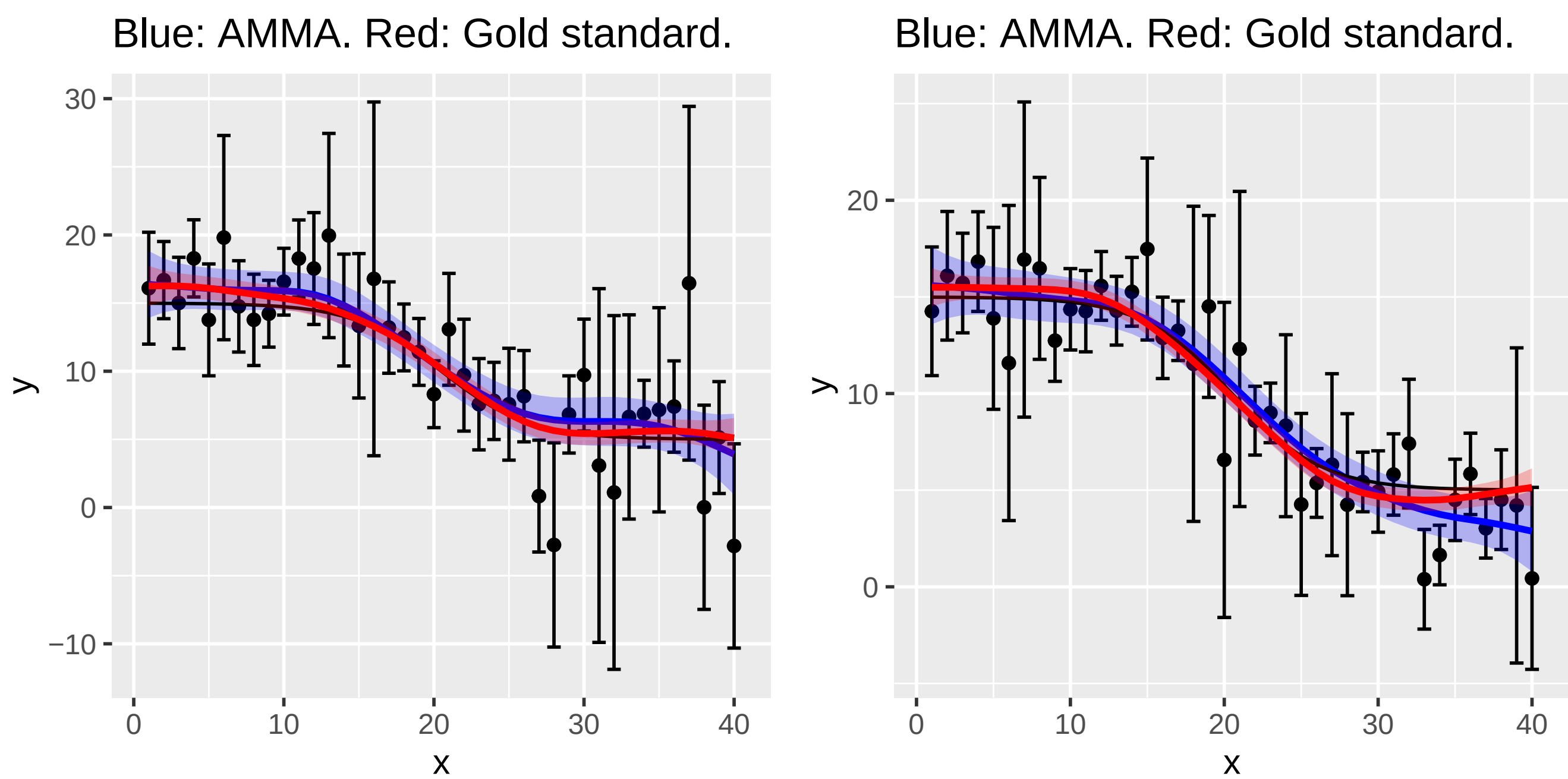

Figure S6: Examples with "less accurate" (c) first-stage errors and random ("R") first-stage sampling effort, comparing AMMA to gold standard.

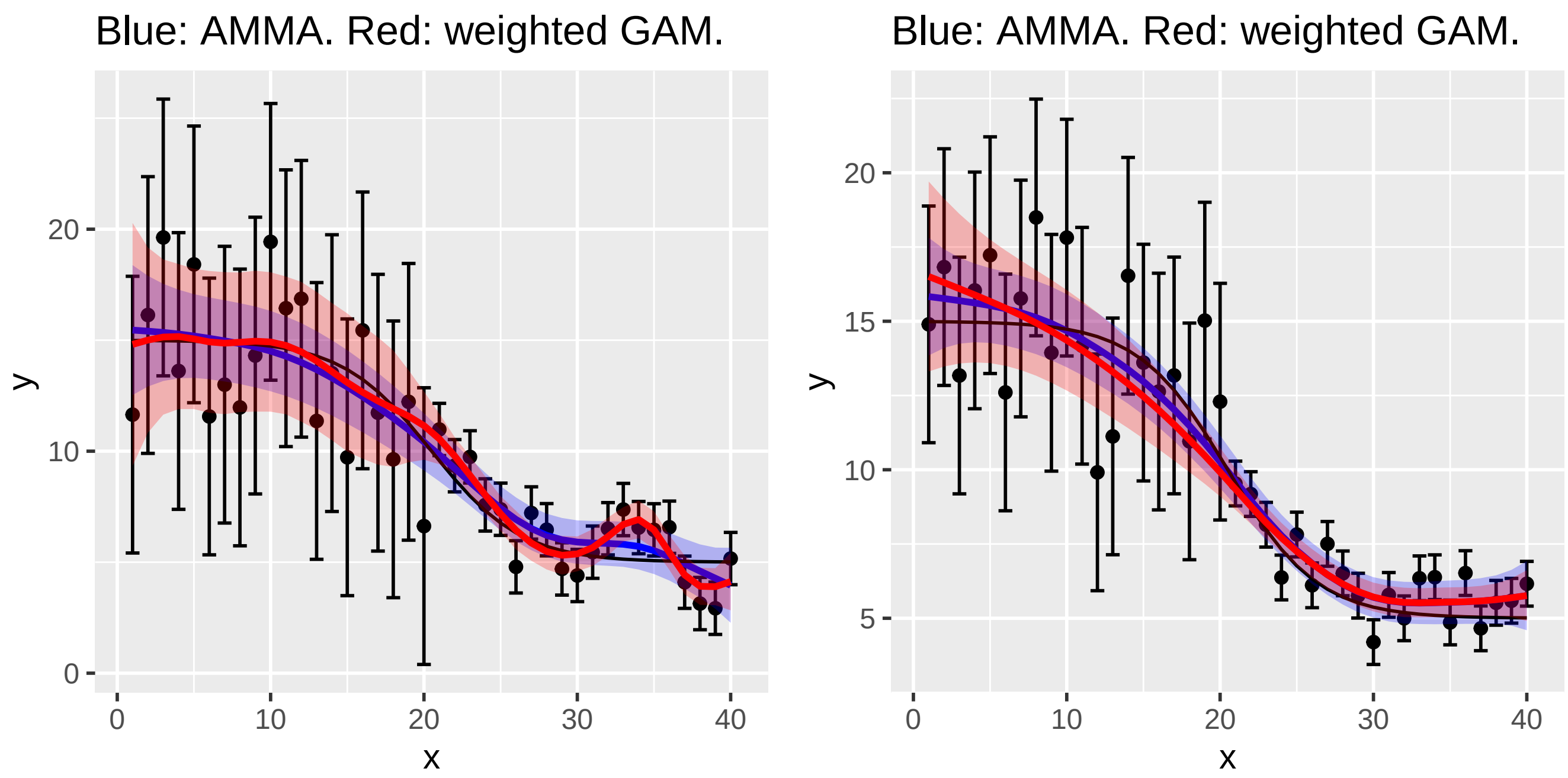

Figure S7: Examples with "more accurate" (c) first-stage errors and step-change ("SC") first-stage sampling effort, comparing AMMA to weighted GAM.

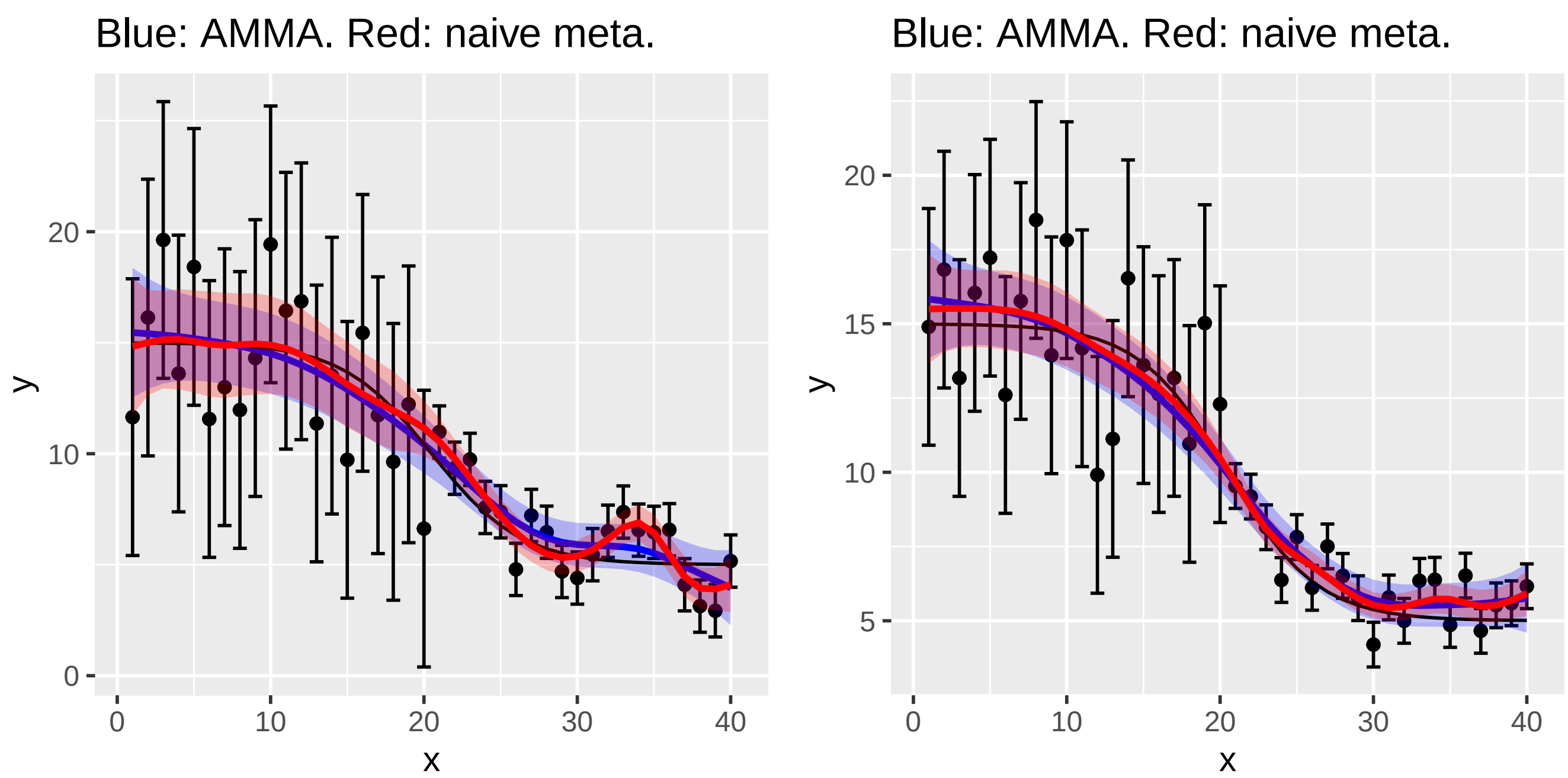

Figure S8: Examples with "more accurate" (c) first-stage errors and step-change ("SC") first-stage sampling effort, comparing AMMA to naive meta-analysis.

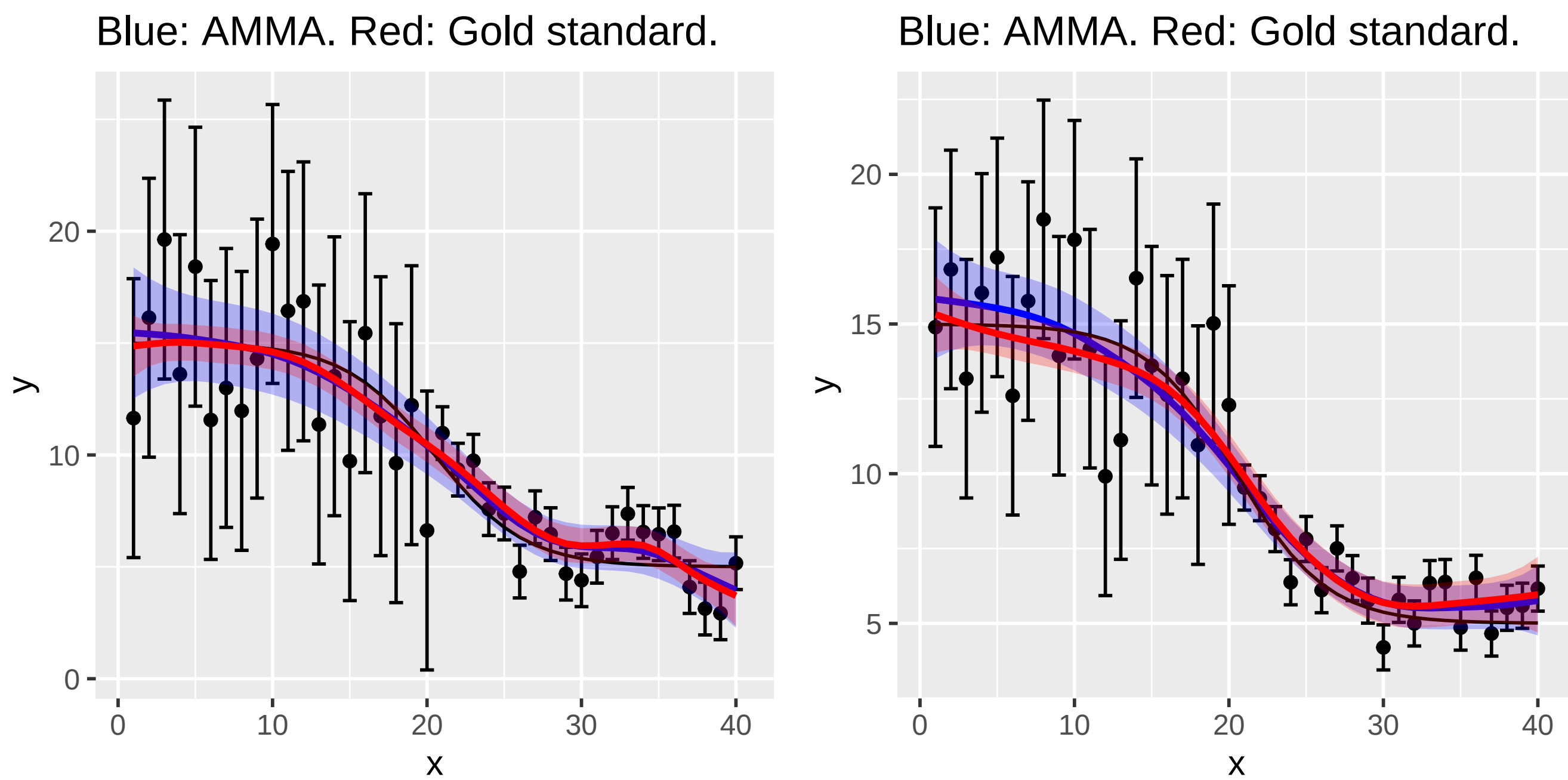

Figure S9: Examples with "more accurate" (c) first-stage errors and step-change ("SC") first-stage sampling effort, comparing AMMA to gold standard.

S6 First-stage analysis of San Francisco Bay water quality data

In this supplement section we describe the steps of obtaining a seasonal mean and standard error from the raw data while accounting for temporal non-independence with a smoothing spline. See e.g. Wood (2017) for details.

Define $\mathbf{c} = (c_1, \ldots, c_I)$ to be the vector of measured chl-a levels at sampling days $t_1, \ldots, t_I$, where the t_i are measured in Julian days, counting continuously through all years of the data set. In the first-stage analysis, we estimate a GAM with a spline through the entire study period,

$$c_i = \alpha_0 + f(t_i) + \nu_i, \tag{S7}$$

where α_0 is the intercept, $f(\cdot)$ is a spline such as a thin-plate or cubic regression spline and $\nu_i \sim N(0, \sigma^2)$ are independent, identically distributed residuals. For these data it is feasible and reasonable to fit one spline with many knots through the entire series, which is what we did. Alternatively, it would be reasonable to fit a separate spline to each year of data, possibly including some data before and after the year to avoid inflated uncertainty at the spline ends. The only concern would be correlation in the resulting seasonal averages across years, but that is effectively zero in this study. Although using separate splines would also be computationally simpler and more general in allowing different degrees of smoothness in each year, it was unnecessary in this case study. For the spline through an entire 43-year series, we used 516 knots, (12 knots per year), to accommodate the high wiggliness observed (Figure 6).

Once the spline has been estimated, we have

$$\mathbf{c} = \mathbf{T}\hat{\boldsymbol{\alpha}} + \boldsymbol{\nu} \tag{S8}$$

Here $\mathbf{T}$ is the matrix of spline basis functions evaluated at points $t_1 \ldots t_I$, along with a column of 1s to incorporate the intercept. $\hat{\boldsymbol{\alpha}}$ is the corresponding vector of estimated spline coefficients concatenated with the estimated intercept, $\hat{\alpha_0}$. And $\boldsymbol{\nu} = (\nu_1, \ldots, \nu_I)$ is the residual vector. In addition we have $\hat{\boldsymbol{\Sigma}}_{\boldsymbol{\alpha}}$, the estimated covariance matrix of $\hat{\boldsymbol{\alpha}}$, produced by the spline estimation methods. In general, one can expect

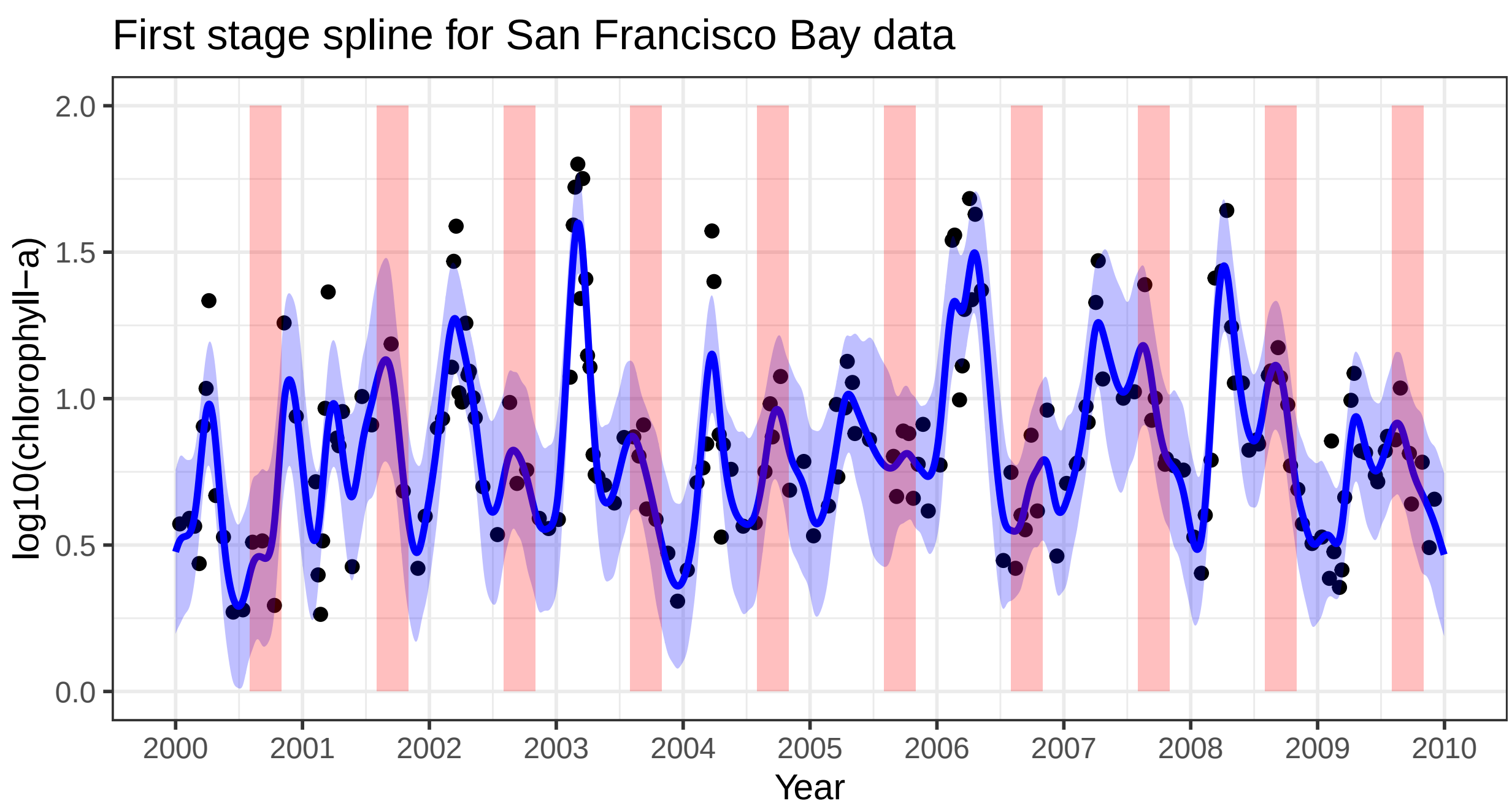

Figure S10: Ten years of chlorophyll-a data from station 30, with a fitted spline and its 95% confidence interval (± 2 standard errors, blue). Red vertical bands show the August-October ("fall") period in each year. The first stage of analysis produces the mean and standard error from the spline of each year's fall.

that first-stage analysis will produce standard errors for scalar estimands and a covariance matrix for vector estimands.

The seasonal average for a predefined window of interest (here Julian day-of-year 213-304 for Aug-Oct), using for example a daily time-scale for averaging, can be obtained by

$$y = \mathbf{A}\mathbf{T}_{\text{pred}}\hat{\boldsymbol{\alpha}} \tag{S9}$$

where $\mathbf{T}_{\text{pred}}$ is the matrix of basis functions evaluated at the set of prediction times (e.g. every day in the time window) and $\mathbf{A}$ is a one-row vector with all entries equal to $1/n_{\text{pred}}$, where n_{pred} is the number of days in the prediction window. Thus $\mathbf{T}_{\text{pred}}\hat{\boldsymbol{\alpha}}$ is a one-column matrix of daily predictions and $\mathbf{A}\mathbf{T}_{\text{pred}}\hat{\boldsymbol{\alpha}}$ is the average of the daily predictions. The variance of the estimate is then the scalar

$$s = \mathbf{A}\mathbf{T}_{\text{pred}}\hat{\boldsymbol{\Sigma}}_{\boldsymbol{\alpha}}\mathbf{T}'_{\text{pred}}\mathbf{A}'. \tag{S10}$$

The choice of a daily time-step for predictions is arbitrary but intuitive and, moreover, sufficiently accurate in the sense that a smaller time-step would not noticeably change results. Alternatively one could integrate the basis functions over time intervals, but the discretized approach here has the merits of simplicity and using the basis functions in an automated manner without need for further knowledge of them. Note that the exact number and date of sampling occasions varies from year to year, so the spline both accommodates different sampling schedules each year and gives standard errors that incorporate the temporal correlation in the data.

The above steps of averaging and determining the sample variance of the average are done for the same time window of each year, for which we append subscript t for $t = 1...m$. This results in $\mathbf{y} = (y_1, \ldots, y_m)$ and $\mathbf{s} = (s_1, \ldots, s_m)$ for use in the AMMA model.

For the first-stage analysis, we used package `wqtrends` (Beck et al., 2026, 2022), which used thin-plate splines. For the second-stage splines in package `splinemixmeta`, we chose cubic regression splines.

S7 AMMA models for second-stage analysis of San Francisco Bay water quality data

The AMMA models for the San Francisco Bay water quality examples used cubic regression splines with 30 knots. The R code for these fits was:

```
splinemixmeta::splinemixmeta(s(x, bs = "cr", k = 30), y ~ year,
se = se, manual_fixed=TRUE)
```

References

Beck, M., P. de Valpine, R. Murphy, I. Wren, A. Chelsky, M. Foley, and D. Senn (2026). *wqtrends: Assess Water Quality Trends with Generalized Additive Models*. version 1.5.2, https://cran.r-project.org/web/packages/wqtrends/index.html. DOI: 10.5281/zenodo.18223524.

Beck, M. W., P. de Valpine, R. Murphy, I. Wren, A. Chelsky, M. Foley, and D. B. Senn (2022). Multi-scale trend analysis of water quality using error propagation of generalized additive models. *Science of The Total Environment 802*, 149927.

de Valpine, P. (2026). *Code and data for "Additive Mixed Meta-Analysis with Spline Meta-Regression for Nonlinear Ecological Trends"*. DOI: 10.5281/zenodo.19404095.

de Valpine, P. and M. Beck (2026). *splinemixmeta: Additive Mixed Meta-Analysis with Spline Meta-Regression*. version 1.0.1, https://CRAN.R-project.org/package=splinemixmeta. DOI: 10.5281/zenodo.19402410.

Gasparrini, A. and F. Sera (2025). *mixmeta: An Extended Mixed-Effects Framework for Meta-Analysis*. version 1.2.2, https://cran.r-project.org/web/packages/mixmeta/index.html.

Marra, G. and S. N. Wood (2011). Practical variable selection for generalized additive models. *Computational Statistics & Data Analysis 55*(7), 2372–2387.

Wood, S. (2025). *mgcv: Mixed GAM Computation Vehicle with Automatic Smoothness Estimation*. Version 1.9-1. https://CRAN.R-project.org/package=mgcv.

Wood, S. N. (2017). *Generalized Additive Models: An Introduction with R* (2 ed.). New York: Chapman and Hall/CRC.
